# Mitochondrial Genome Comparison and Phylogenetic Analysis of the Family Carabidae (Coleoptera: Adephaga)

**DOI:** 10.3390/biology15181593

**Published:** 2026-09-09

**Authors:** Jinyu Zhan, Pingzhou Zhu, Kaixuan Liu, Rongrong Shen, Hongbin Liang, Xinpu Wang, Ming Bai

**Affiliations:** 1 School of Forestry and Grassland Science, Ningxia University, Yinchuan 750021, China; 2 State Key Laboratory of Animal Biodiversity Conservation and Integrated Pest Management, Institute of Zoology, Chinese Academy of Sciences, Beijing 100101, China; 3 State Key Laboratory of Agricultural and Forestry Biosecurity, MOA Key Lab of Pest Monitoring and Green Management, College of Plant Protection, China Agricultural University, Beijing 100101, China; 4 Hebei Collaborative Innovation Center for Eco-Environment, College of Life Sciences, Hebei Normal University, Shijiazhuang 050024, China; 5 School of Agriculture, Ningxia University, Yinchuan 750021, China; 6 Hebei Key Laboratory of Animal Diversity, College of Life Science, Langfang Normal University, Langfang 065000, China

**Keywords:** ground beetles, mitogenome, genomic characterization, molecular phylogeny

## Abstract

The family Carabidae, one of the largest families within Coleoptera, represents one of the most important groups of predatory insects and bioindicators in ecosystems, playing significant roles in agricultural pest control and ecological environment assessment. However, the phylogenetic relationships within this family remain poorly resolved, and publicly available mitochondrial genome data remain limited. In this study, we employed high-throughput sequencing to determine and report for the first time the complete mitogenomes of four carabid species—*Poecilus fortipes* (Chaudoir, 1850) and *Poecilus gebleri* (Dejean, 1828) from the subfamily Pterostichinae, and *Pristosia nitidula* (A. Morawitz, 1862) and *Cephalosdrophus marinae* (Lassalle & Marcilhac, 1999) from the subfamily Platyninae—as well as to resequence *Carabus brandti* Faldermann, 1835 from the subfamily Carabinae. Using 124 carabid mitochondrial genome sequences obtained from NCBI, this study reconstructed the phylogenetic relationships among 21 subfamilies within Carabidae. The results showed that the sizes of mitochondrial genomes ranged from 15,800 to 17,352 bp in length and contained 37 typical genes and a control region. Combined with previously reported mitogenomic data, we found all protein-coding genes (PCGs) initiated with standard start codons ATN or TTG and ended with TAN or an incomplete stop codon single T. Evolutionary rate analysis (Ka/Ks) revealed *ATP8* was the fastest-evolving gene, whereas *COX1* was the slowest. Except for tRNA-*Ser* (AGN), whose DHU arm simply formed a loop, and tRNA-*Phe* (*C. marinae*), whose TΨC loop was absent, the rest of the tRNA sequences could be folded into a typical cloverleaf secondary structure. Our results suggest that the phylogenetic position and taxonomic status of Cicindelinae remain unresolved, with alternative placements recovered depending on the dataset analyzed. The monophyly of most carabid subfamilies was confirmed, with the exception of Brachininae, Trechinae, Licininae, and Platyninae. The findings of this study provide valuable insights into carabid phylogeny. Further refinement of the phylogenetic relationships within the family can be achieved by additional nuclear markers, broader taxon sampling, and complementary phylogenomic datasets.

## Simple Summary

This study enhances our understanding of the evolutionary relationships within the ground beetle family Carabidae by sequencing the complete mitochondrial genomes of five species, including four newly determined mitogenomes and one resequenced species. These mitogenomes contain the typical set of 37 genes and a control region, with genome sizes ranging from 15,800 to 17,352 bp. Our analyses reveal that most mitochondrial protein-coding genes exhibit conserved start and stop codon patterns, while ATP8 evolves the fastest and *COX1* the slowest among these genes. The majority of tRNAs form the typical cloverleaf structure, with a few structural variations observed in tRNA-*Ser* (AGN) and tRNA-*Phe*. By combining our dataset with 124 previously published carabid mitogenomes, we reconstructed the phylogenetic relationships of 21 subfamilies within Carabidae. Our results support the monophyly of most subfamilies, but also identify non-monophyletic conditions for Brachininae, Trechinae, Licininae, and Platyninae. The phylogenetic position of Cicindelinae also remains ambiguous, with alternative topological placements recovered under different dataset treatments. These findings provide new mitochondrial genomic evidence for elucidating the evolutionary history of Carabidae and underscore the necessity of broader taxonomic sampling, as well as the integration of nuclear genomic data, to further resolve the contentious relationships within this diverse beetle family.

## 1. Introduction

Ground beetles (Carabidae) represent one of the most species-rich families within the order Coleoptera, and are among the most widely distributed lineages in the suborder Adephaga. Globally, approximately 42,000 species have been described, belonging to 36 subfamilies and 89 tribes (Insecta in Catalogue of Life (COL) (10 October 2025) [1]. While many species retain well-developed hind wings and the ability to fly, the majority inhabit soil and surface substrates, with a minority found on trees or on herbaceous plants. Therefore, they are often referred to as “ground-dwelling beetles”. Ground beetles exhibit broad feeding habits. Although most are predatory, significant omnivorous and herbivorous groups also exist [2,3,4]. Their survival depends on a combination of biotic and abiotic factors characteristic of specific geographic or natural regions. As important natural enemies and ecological indicators, they play significant roles in agricultural pest control and environmental assessment [5,6,7]. Owing to their diverse habitats, high species richness, remarkable adaptability, and ease of collection, ground beetles have become a focal group in entomological and ecological research [8,9,10].

Although the phylogeny of carabid beetles has been extensively studied using both morphological and molecular data, their relationships remain contentious. Traditional taxonomic studies have relied on a variety of morphological characters, including female genitalia [11], wing structures [12,13], muscular systems [14,15], defensive glands and secretions [16,17], and larval morphology [18,19,20,21]. Although these studies have resolved the classification of certain groups, they have not conclusively established the monophyly of Carabidae nor reached a consensus on many aspects of tribal-level relationships [22,23,24,25]. With the advancement of molecular techniques, genetic data have been increasingly employed in beetle phylogenetic studies. Maddison et al. [26] conducted an early phylogenetic analysis of Carabidae using *18S* ribosomal DNA (*18S* rDNA), a slowly evolving nuclear ribosomal marker commonly used for resolving higher-level phylogenetic relationships. Their study provided insights into the placement of several tribes and suggested close affinities among Cicindelinae, Rhysodinae, Paussinae, and Harpalinae. Nevertheless, the phylogenetic positions of many carabid lineages remained unresolved. Subsequent studies incorporating multi-gene datasets have improved the resolution of carabid phylogenetic relationships. Bocak et al. [27] found that Carabidae was monophyletic based on a supermatrix dataset including *18S* rRNA, *28S* ribosomal RNA (*28S* rRNA), mitochondrial large subunit ribosomal RNA (*rrnL*)*,* and cytochrome c oxidase subunit I (*COX1*). Notably, Gough et al. [28] recovered Cicindelinae and Rhysodinae as sister groups based on six protein-coding genes and three ribosomal gene fragments. Choi et al. [29] revealed that the subfamily Harpalinae was paraphyletic with respect to Brachininae.

The insect mitochondrial genome is a circular, double-stranded covalently closed DNA molecule, typically ranging from 14 to 20 kb in size. It contains 37 highly conserved genes, i.e., 13 protein-coding genes (PCGs), 22 transfer RNA genes (tRNAs), and two ribosomal RNA genes (rRNAs). Additionally, it contains an A + T-rich control region [30]. Owing to its relatively small molecular size, simple structure, high copy number, conserved gene arrangement, rapid evolutionary rate, and predominantly maternal inheritance in most animals with little intraspecies recombination, mtDNA has been widely used in species identification, phylogenetics, and evolutionary studies [31,32]. Certain conserved mitochondrial genes, such as *cox1*, *cox2*, and *cytb*, are frequently employed to investigate phylogenetic relationships at lower taxonomic levels [33,34]. With advances in molecular biology and genomics, there has been a growing effort to reconstruct higher-level phylogenies using nuclear genes, mitochondrial genomes, and ultraconserved elements (UCEs) [35,36,37,38,39]. Despite the high species diversity of Carabidae, mitochondrial genomic resources available in public databases remain limited. As of November 2025, only 230 mitochondrial genome entries representing 145 species, including both complete and partial sequences, were available in the GenBank, accounting for approximately 0.35% of the currently described species diversity. This substantial gap in mitochondrial genomic sampling limits comprehensive assessments of mitochondrial genome evolution and hinders a more robust understanding of phylogenetic relationships within Carabidae. Although mitochondrial genomic datasets have been increasingly applied to investigate carabid phylogeny, studies based on mitochondrial genomic data by Li et al. [40], Raupach et al. [41], Lin et al. [42], and Zhu et al. [43] have not consistently recovered Carabidae as monophyletic, indicating the need for broader taxon sampling and additional genomic evidence.

Therefore, expanding mitochondrial genomic resources for representative taxa within Carabidae is important for providing additional evidence toward resolving phylogenetic relationships within the family. In this study, the mitochondrial genomes of five carabid beetles belonging to four genera across three subfamilies were sequenced or resequenced, including *Poecilus fortipes* (Chaudoir, 1850) and *Poecilus gebleri* (Dejean, 1828) from Pterostichinae, *Carabus brandti* Faldermann, 1835 from Carabinae, as well as *Pristosia nitidula* (Morawitz, 1862) and *Cephalosdrophus marinae* (Lassalle & Marcilhac, 1999) from Platyninae. Specifically, we aimed to (1) characterize the mitochondrial genomic features of these species, including genome organization, nucleotide composition, codon usage bias, and tRNA secondary structures; and (2) reconstruct phylogenetic relationships within Carabidae by integrating the newly obtained mitogenomes with other available mitochondrial genomes from GenBank using maximum likelihood and Bayesian inference methods based on different datasets. The results provide additional mitochondrial genomic resources for Carabidae and contribute to a better understanding of mitochondrial genome evolution and phylogenetic relationships within this family. These resources may serve as valuable genetic references for future ecological and biodiversity studies, including species identification, community assessment, and the evaluation of carabid beetles as natural enemies and bioindicators.

## 2. Materials and Methods

### 2.1. Sampling, DNA Extraction and Sequencing

Adult specimens of five carabid beetles used in this study were collected in Helan Mountain, Yinchuan, Ningxia, China, using pitfall traps (Table 1). The specimens were preserved in 100% ethanol immediately in the field and stored at −20 °C before DNA extraction. All specimens were deposited in the National Animal Collection Resource Center, Institute of Zoology, Chinese Academy of Sciences.

A single adult specimen from each of the five carabid species was selected for DNA extraction in this study. Total genomic DNA was individually extracted from each specimen using muscle tissues of meso- or metathorax or hind legs using the sodium dodecyl sulfate (SDS) method combined with column adsorption for DNA purification. These tissues are mitochondria-rich, ensuring high mtDNA yield with minimal digestive tract contamination. DNA libraries with an average insert size of 350 bp in length were prepared using Hieff NGS^®^ OnePot Pro DNA Library Prep Kit (Yeasen Biotechnology Co., Ltd., Shanghai, China) and sequenced on a DNBSEQ-T7 platform (Benagen, Wuhan, China) using the paired-end 150 bp (PE150) strategy.

### 2.2. Mitochondrial Genome Assembly and Annotation

Reference-guided assembly and annotation were performed with Geneious Prime version 2025.0.2 [44]. The published mitogenome of *Abroscelis anchoralis* (GenBank accession number: NC_038191), *Carabus changeonleei* (GenBank accession number: NC_046469), and *Calathus gomerensis* (GenBank accession number: OQ716325) were used as the reference sequences in the study. The MITOS2 tool on Galaxy Server version 24.1.2 (https://usegalaxy.org/) (accessed on 17 August 2025) and tRNAscan-SE version 2.0 Web Server (https://trna.ucsc.edu/tRNAscan-SE/) (accessed on 17 August 2025) were used to annotate the tRNAs and predict their secondary structure models [45,46]. Subsequently, the secondary structures of tRNA genes were visualized using Adobe Illustrator 2024. The online visualization software Proksee (https://proksee.ca/) (accessed on 17 August 2025) was utilized to visualize the complete mitochondrial genome [47]. The newly sequenced mitogenomes were deposited in GenBank with the accession numbers PX606622–PX606626.

The nucleotide composition and relative synonymous codon usage (RSCU) were calculated using PhyloSuite version 2.0 [48]. The AT-skew and GC-skew were calculated as follows: AT-skew = (A − T)/(A + T) and GC-skew = (G − C)/(G + C) [49]. The RSCU figure was drawn using the packages including ggplot2 version 3.5.1 and aplot version 0.2.3 in R version 4.4.1. DnaSP version 6.0 was used to calculate nonsynonymous substitution rates (Ka), which represent nucleotide substitutions causing amino acid changes, synonymous substitution rates (Ks), which represent substitutions that do not result in amino acid changes, and Ka/Ks ratios for the 13 protein-coding genes [50].

### 2.3. Phylogenetic Analyses

For phylogenetic analyses, a total of 129 mitogenomes of Carabidae (including five obtained in this study and 124 downloaded from GenBank) (Table S1) were used as ingroups. The mitochondrial genome of *Trachypachus holmbergi* (Adephaga: Trachypachidae) (GenBank accession number: NC_011329.1) was selected as the outgroup. Phylogenetic analyses were performed using four different datasets: (1) PCGsAA, comprising amino acid sequences of the 13 PCGs; (2) PCGsNT, comprising nucleotide sequences of the 13 PCGs; (3) PCGs123R, comprising 13 PCGs (all codon positions) plus two ribosomal RNA genes (*12S* and *16S*); (4) PCGs12R, comprising 13 PCGs (with the third codon positions removed) plus two ribosomal RNA genes (*12S* and *16S*). The 22 tRNAs were not included in the phylogenetic analyses because they are relatively short and highly conserved, often providing insufficient signal for resolving deep-level nodes compared to longer PCGs and rRNAs.

PCGs, tRNAs and rRNAs were retrieved, aligned, trimmed using Gblocks, and concatenated in PhyloSuite version 2.0 [48]. Multiple sequence alignment of the protein-coding genes was performed using MAFFT version 7.526 [51]. The alignments were refined with Gblocks version 0.91b [52] and trimAI [53] to remove poorly aligned regions and positions of ambiguous homology. The concatenation of alignments into four datasets was implemented in PhyloSuite v2.0 [48]. Subsequently, the optimal partitioning scheme and substitution model were determined with PartitionFinder version 2.0 [54] for Maximum likelihood (ML) and Bayesian inference (BI) analyses.

We conducted ML analyses in IQ-TREE version 2.2.2.6 [55] for our concatenated datasets with the partition model (“-p”) [56]. The concatenated matrices were partitioned by locus, and the best partitioning schemes and substitution models were selected using ModelFinder (“-MFP”) under the Bayesian Information Criterion (“-merit BIC”) Nodal support was computed with 1000 ultrafast bootstraps (UFBoot; “-bb” option) replicates and 1000 SH-aLRT (“-alrt”) replicates [57,58,59]. And the Bayesian inference (BI) were conducted using MrBayes version 3.2.6 [60]. Outputs were visualized and edited using Figtree version 1.4.4 [61].

## 3. Results

### 3.1. Characteristics of the Mitogenomes of the Five Carabid Beetles

**Figure 1:**
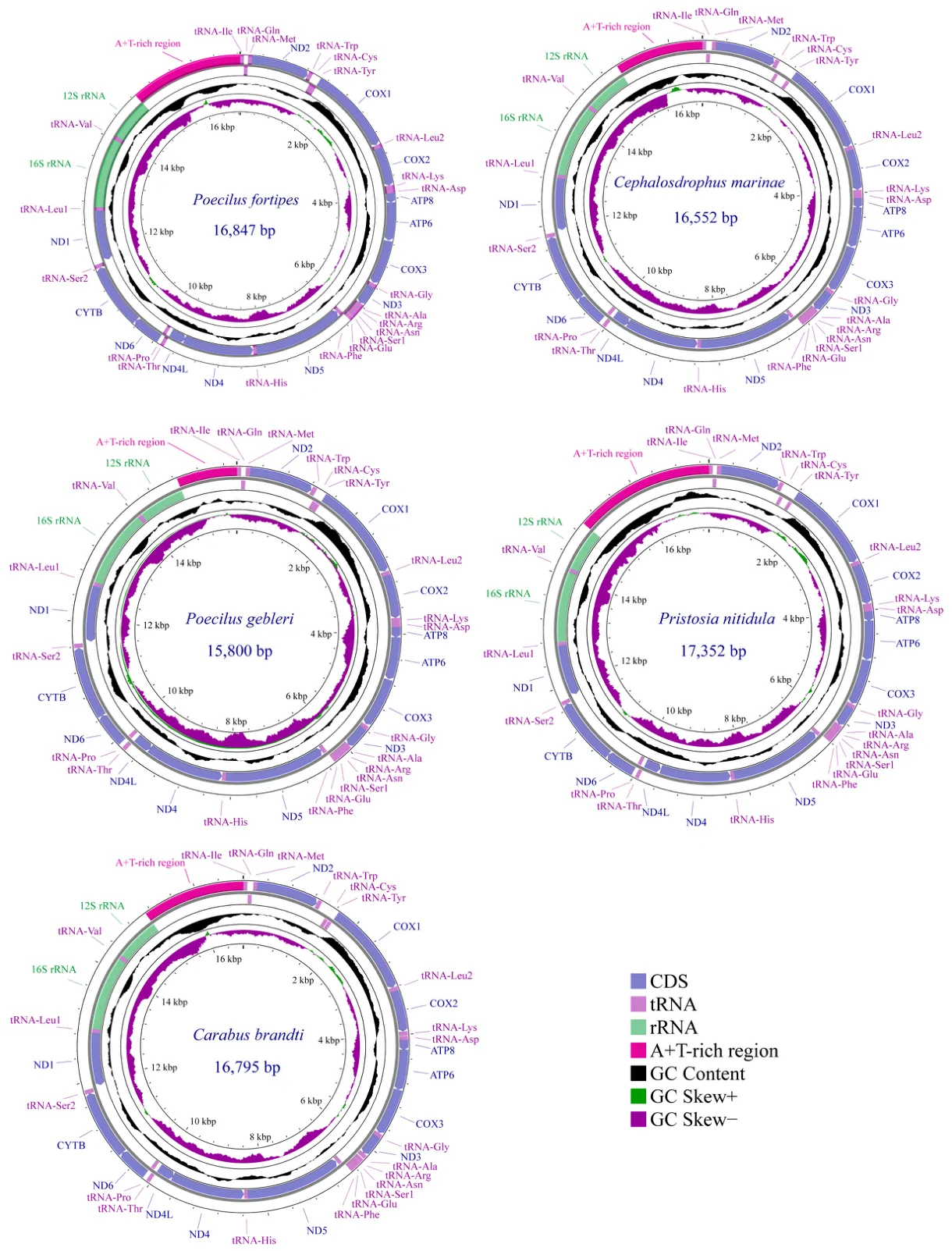
Circular maps of five newly sequenced mitogenomes of Carabidae.

The mitogenomes of *Poecilus fortipes* (16,847 bp), *Poecilus gebleri* (15,800 bp), *Carabus brandti* (16,795 bp), *Pristosia nitidula* (17,352 bp), and *Cephalosdrophus marinae* (16,552 bp) all showed a typical double-stranded circular structure (Figure 1). The mitochondrial genome sequence lengths of these five species fell within the range of existing ground beetle mitochondrial genome sequences (12,639 bp–21,161 bp) in the GenBank database. These newly sequenced mitogenomes all contained 37 typical genes, with 13 PCGs, two rRNA genes, 22 tRNA genes, and an A + T-rich region. Four of the 13 PCGs (*ND5*, *ND4*, *ND4L*, and *ND1*), eight tRNAs (*trnQ*, *trnC*, *trnY*, *trnF*, *trnH*, *trnP*, *trnL* (CUN), and *trnV*) and two rRNAs (*rrnL* and *rrnS*) were encoded on the minority-strand (N-strand), while the remaining 23 genes were encoded on the majority-strand (J-strand).

**Figure 2:**
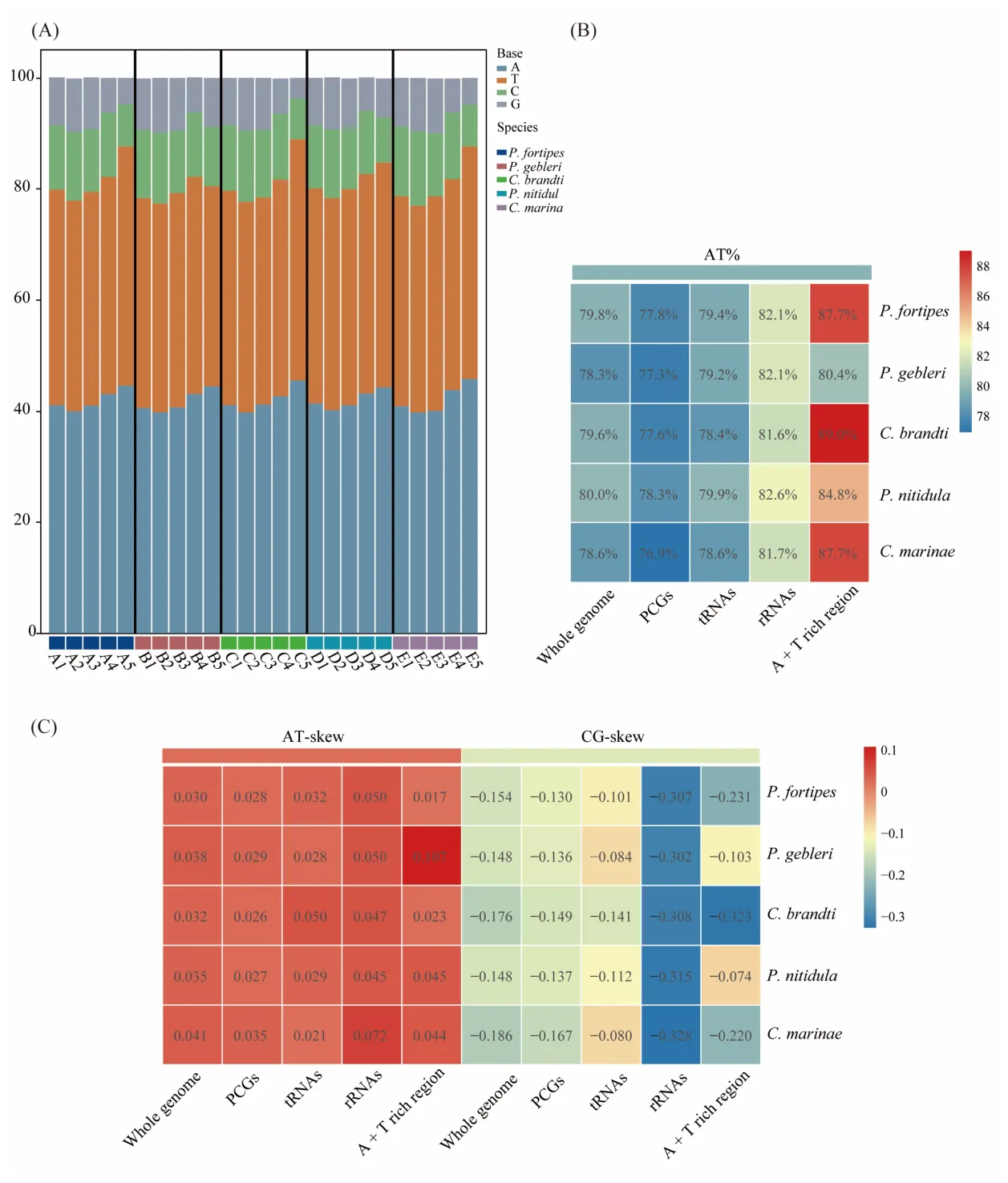
Nucleotide composition of various datasets of five carabid beetles. (**A**) Base composition; (**B**) the A + T content; (**C**) the AT-skew and GC-skew. Note: A1. B1, C1, D1 and E1 represent the whole genome; A2, B2, C2, D2 and E2 represent PCGs; A3, B3, C3, D3 and E3 represent tRNAs; A4, B4, C4, D4 and E4 represent rRNAs; A5, B5, C5, D5 and E5 represent A + T rich region.

The nucleotide compositions of the five mitogenomes are summarized in Figure 2A. The base composition was A (41.1%), T (38.7%), C (11.7%), G (8.6%) in *Poecilus fortipes*, A (40.6%), T (37.7%), C (12.4%), G (9.2%) in *Poecilus gebleri*, A (41.1%), T (38.5%), C (12.0%), G (8.4%) in *Carabus brandti*, A (41.4%), T (38.6%), C (11.5%), G (8.9%) in *Pristosia nitidula*, and A (40.9%), T (37.7%), C (12.7%), G (8.7%) in *Cephalosdrophus marinae*, with a total A + T content of 79.8%, 78.3%, 79.6%, 80.0%, and 78.6%, respectively (Figure 2B). The AT-rich codon usage in the five carabid beetle species was consistent with the overall base composition bias of insect mitochondrial genomes. The AT-skew values were positive and the GC-skew values were negative across the whole mitogenome, indicating a preference for the use of A and C bases over T and G bases. The pattern was also observed in the mitochondrial genomes of species belonging to the subfamilies Pterostichinae, Carabinae and Platyninae sequenced in this study (Figure 2C).

### 3.2. PCGs and Codon Usage

Annotations of the five newly sequenced mitogenomes are provided in Table S2, and summaries of the genes comprising these mitogenomes are presented in Table S5. The total lengths of 13 PCGs in all mitogenomes of these beetles were 11,212 bp, 11,195 bp, 11,222 bp, 11,205 bp, and 12,597 bp in order. In line with other insects of Coleoptera, the PCGs in the mitochondrial genomes of these five carabid species demonstrated a distinct bias toward adenine and thymine in their base composition. Specifically, the AT content was highest in *Pristosia nitidula* (78.3%) and lowest in *Cephalosdrophus marinae* (76.9%) among the groups (Figure 2B). Analysis of skewness revealed that, across all sequenced species, a higher prevalence of A relative to T and a lower abundance of G relative to C were observed, with the AT-skew being positive (ranging from 0.026 to 0.035), and the GC-skew uniformly negative (ranging from −0.167 to −0.130) (Figure 2C).

Most PCGs in the five carabid species used the conventional ATN (ATA/T/C/G) start codons, except for the *ND1* gene, which starts with the unconventional TTG (Table S2). The variation between an incomplete “T” and a complete “TAA” stop codon is a result of post-transcriptional polyadenylation, where the addition of “AA” creates a functional stop signal—a common feature in insect mitogenomes. *COX1* and *COX2* applied the incomplete termination codon T as the stop codon. Most of the remaining PCGs terminated with TAA or TAG, except for the *ND5* and *ND4* genes. The termination codons used in the *ND5* gene differ among the five species, and the same is true for the *ND4* gene as well. The incomplete termination codon T is consistently used in the *ND5* gene of four species (*Poecilus fortipes*, *Poecilus gebleri*, *Pristosia nitidula*, and *Cephalosdrophus marinae*) and the *ND4* gene of two species of *Poecilus*, while TAA is employed as the stop codon in *ND5* gene of *Carabus brandti* and *ND4* gene of the remaining three species (*C. brandti*, *P. nitidula*, and *C. marinae*).

**Figure 3:**
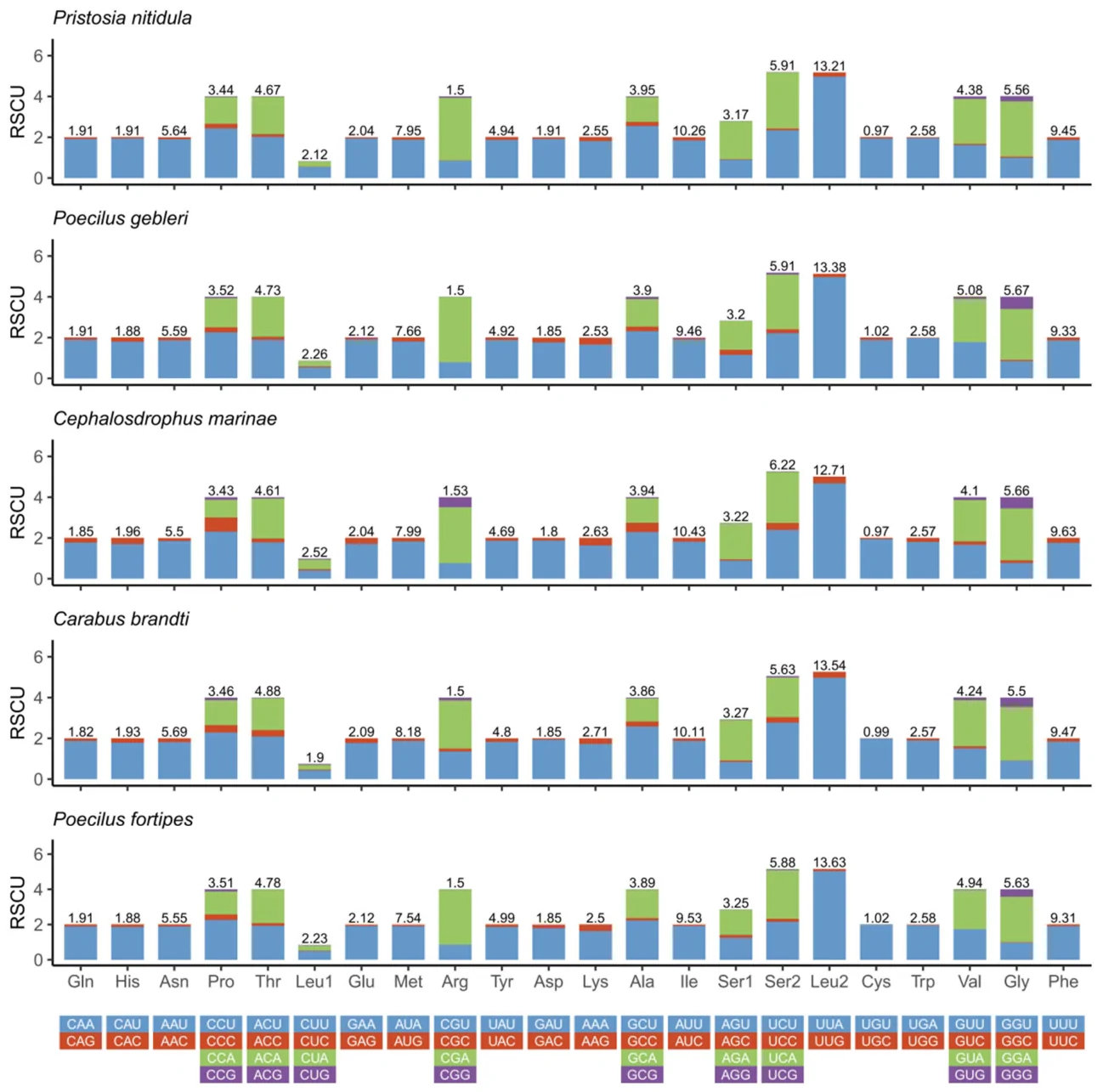
Relative synonymous codon usage (RSCU) in the PCGs of the five newly sequenced mitogenomes of Carabidae (codon families are labeled on the x-axis. Values on the top of the bars refer to amino acid usage).

Relative synonymous codon usage (RSCU) values for the five species are given in Figure 3 and Table S3. The 13 protein-coding genes (PCGs) of *Poecilus fortipes*, *Carabus brandti*, *Cephalosdrophus marinae*, *Poecilus gebleri*, and *Pristosia nitidula* contained a total of 3736, 3740, 3738, 3730, and 3731 codons, respectively. *Leu2* (13.63%, 13.54%, 12.71%, 13.38%, 13.21%), *Ile* (9.53%, 10.11%, 10.43%, 9.46%, 10.26%), and *Phe* (9.31%, 9.47%, 9.63%, 9.33%, 9.45%) were the most frequently encoded amino acids (over 9%) in the PCGs, followed by *Met*, *Ser2*, *Asn*, and *Gly* (Figure 3). UUA-*Leu2* was the most frequently used codon, followed by CGA-*Arg*, UCA/UCU-*Ser1*, GCU-*Ala*, and GGA-*Gly*. The codon usage pattern showed a high frequency of codons composed of A and T bases. Conversely, codons rich in C and G bases generally exhibited relative synonymous codon usage (RSCU) values less than 1.00, indicating lower frequencies of use (Table S3). This reflects a clear AT bias in the mitochondrial genomes of the five carabid beetles.

**Figure 4:**
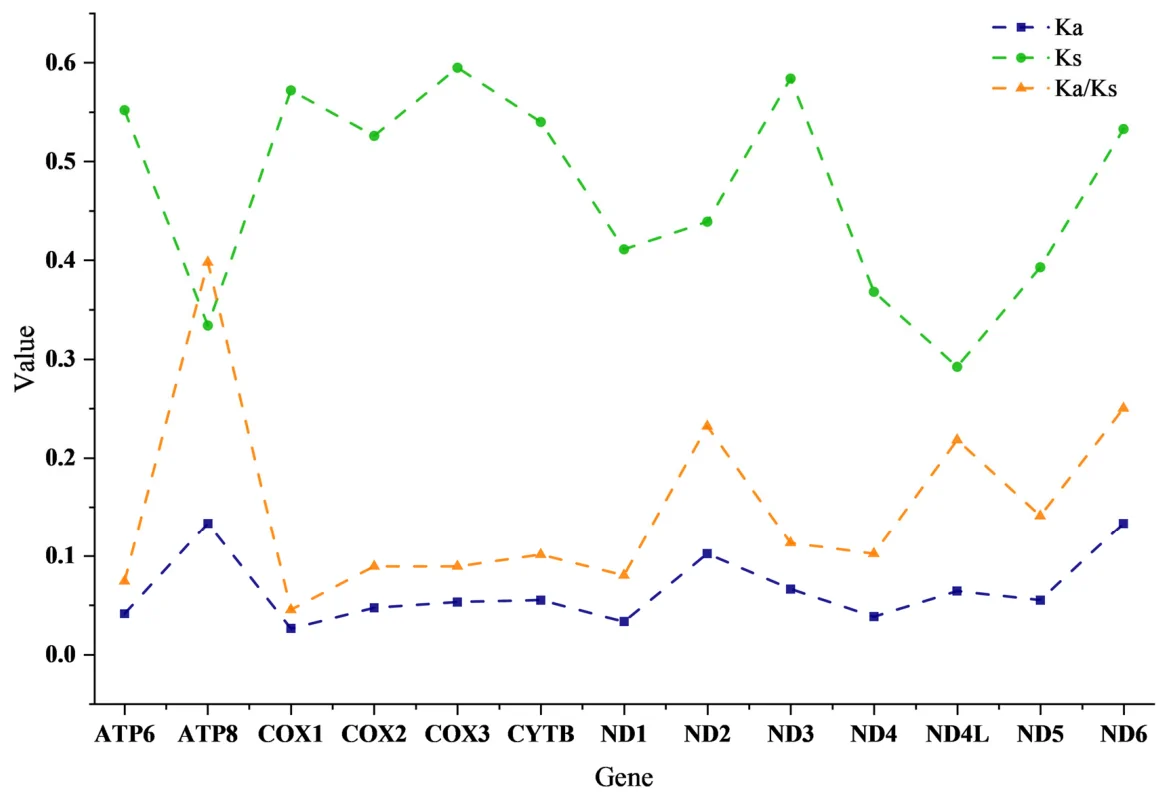
The Ka, Ks, and Ka/Ks ratios of 13 PCGs among five carabid beetles.

The Ka/Ks ratio is commonly used to infer selective pressure acting on PCGs, where values lower than 1 generally indicate purifying selection, values greater than 1 may indicate positive selection, values close to 1 suggest neutral evolution, and when the value is closer to 1, the selection pressure on the gene is smaller [62]. The Ka, Ks, and Ka/Ks ratios for each PCG of the five carabid beetles are shown in Figure 4 and Table S4. All Ka/Ks ratios ranged from 0.045 to 0.398, indicating that all PCGs were under purifying selection. The Ka/Ks value for *ATP8* was the highest, followed by those of *ND6*, *ND2*, *ND4L*, whereas *COX1* exhibited the lowest value. Our results showed that *COX1* (0.045) exhibited the strongest purifying selection and had the lowest evolutionary rate among the 13 PCGs. These results also demonstrated that *COX1* exhibited high sequence conservation and the least genetic variability across the five carabid species examined. *ATP8* is under the least selection pressure and the fastest-evolving gene among the mitochondrial PCGs in *Carabidae*.

### 3.3. tRNAs, rRNAs and Control Region

The total lengths of the 22 tRNAs were 1180 bp, 1177 bp, 1158 bp, 1176 bp and 1154 bp in *Poecilus fortipes*, *Poecilus gebleri*, *Carabus brandti*, *Pristosia nitidula*, and *Cephalosdrophus marinae*, respectively. Individual tRNA genes ranged from 65 bp to 72 bp, 65 bp to 72 bp, 64 bp to 71 bp, 64 bp to 71 bp, and 60 bp to 71 bp, respectively (Table S2). The AT content of tRNAs ranged from 78.4% to 79.9%, which had a strong AT bias (Figure 2B). Among the 22 tRNAs analyzed in all five carabid beetles, positive AT-skew and GC-skew values were observed, indicating a preference for the use of A and G bases.

The predicted secondary structures of tRNAs are shown in Figures S1–S5. The five mitogenomes contained the typical set of 22 tRNAs corresponding to all 20 amino acids. Typically, tRNA secondary structures feature four arms: the amino acid acceptor arm, DHU arm, anticodon arm, and TΨC arm, as well as four loops: the variable loop, DHU loop, anticodon loop, and TΨC loop. Except for tRNA-*Ser* (AGN), whose DHU arm simply formed a loop, and tRNA-*Phe* (*C. marinae*), whose TΨC loop was lost, all remaining tRNAs could be folded into the typical cloverleaf secondary structure. Moreover, U-G mismatched base pairs were found in the tRNA secondary structures of the five ground beetles.

Both rRNA genes were encoded on the N-strand. The *16S* rRNA was located between tRNA-*Leu* (CUN) and tRNA-*Val* with a length of 1309–1336 bp. The *12S* rRNA ranged from 786 bp to 791 bp in length between tRNA-*Val* and the control region, which is consistent with the arrangement order of other insects (Table S2). The AT content of rRNAs ranged from 81.6% to 82.6%, which was the highest among all mitochondrial genes (Figure 2B). The largest non-coding region, located between *12S* rRNA and tRNA-*Ile*, was the control region, which is involved in the regulation of mitochondrial genome replication and transcription.

The length of the control regions ranged from 942 bp to 2393 bp among the five species, with *Pristosia nitidula* having the longest control region, and *Poecilus gebleri* the shortest (Table S2). This result is consistent with the findings regarding mitochondrial genome length in this study. Differences in control region length largely accounted for the variation in total mitogenome size among the five species. The AT content was between 80.4% and 89.0%, presenting a pronounced AT bias (Figure 2B).

### 3.4. Phylogenetic Analysis

**Figure 5:**
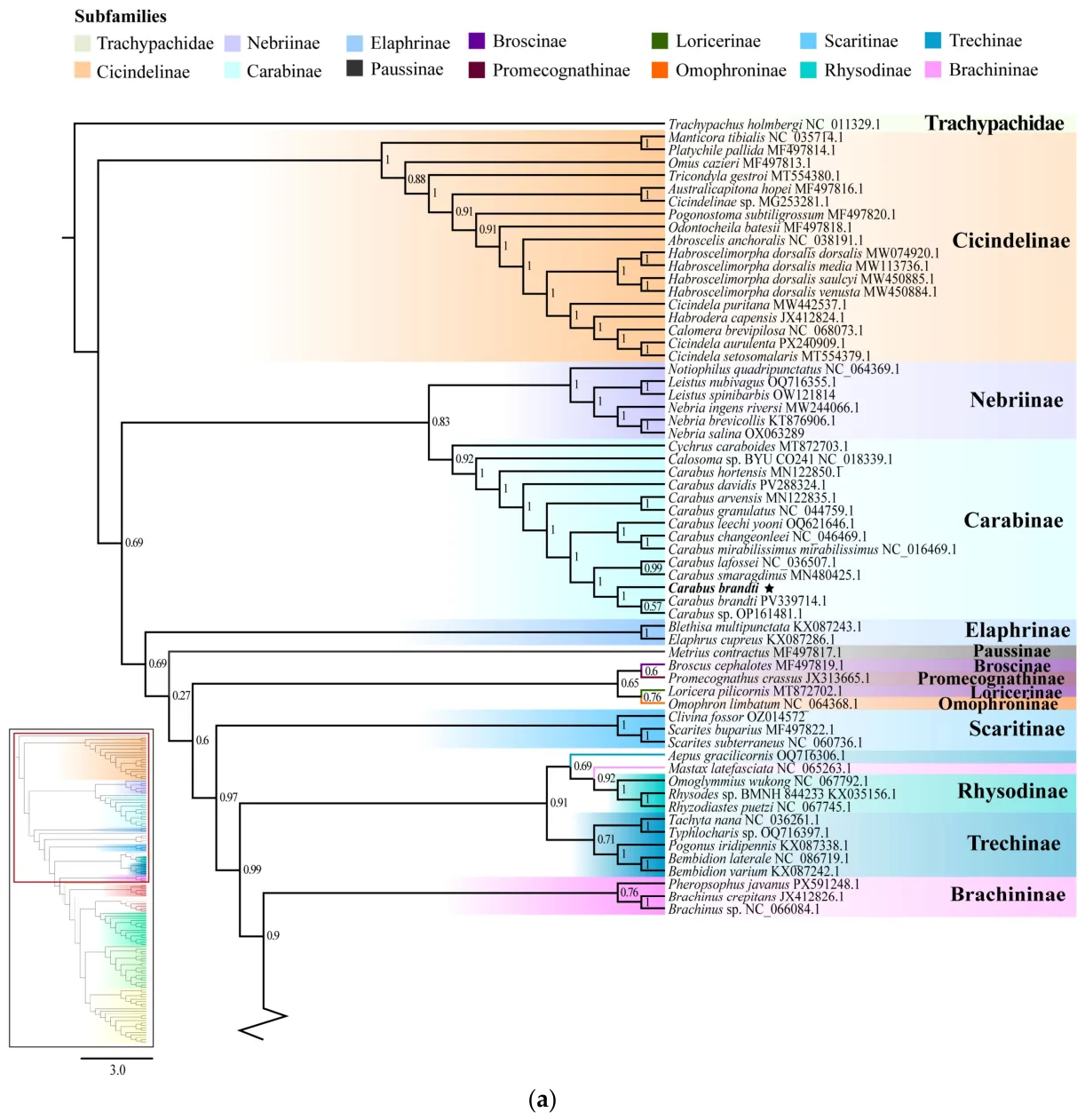

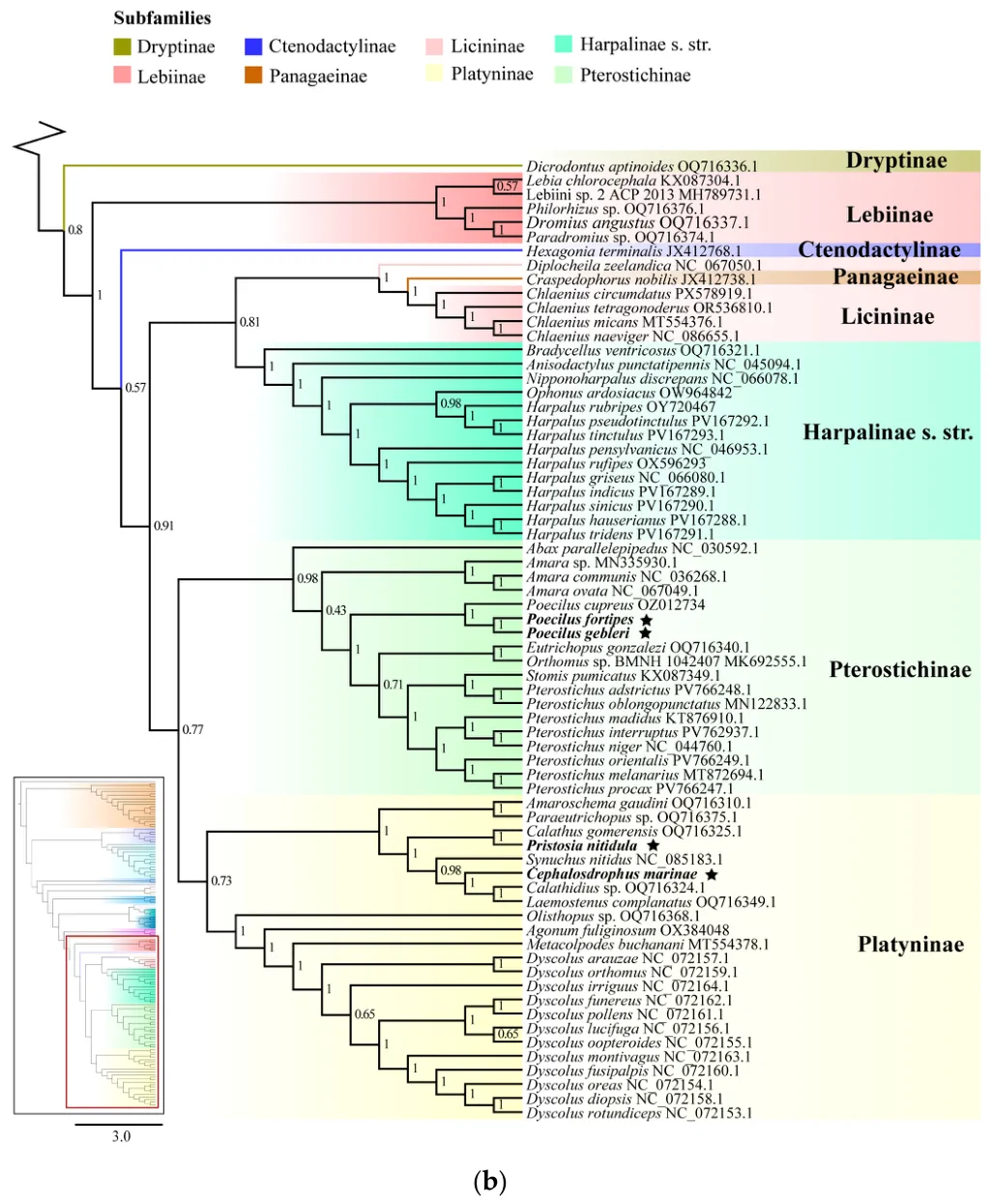
(**a**) Phylogenetic trees of Carabidae inferred using Bayesian inference based on the PCGs123R dataset. The numbers near the nodes are Bayesian posterior probabilities (PPs). Scale bar represents substitutions/site. (**b**) Continuation of phylogenetic trees of Carabidae inferred using Bayesian inference based on the PCGs123R dataset. The numbers near the nodes are Bayesian posterior probabilities (PPs). Scale bar represents substitutions/site. ★ indicates species newly sequenced in this study.

Building upon previous studies, we expanded the sample size of available mitochondrial genome data from three genera (*Poecilus*, *Pristosia*, and *Cephalosdrophus*) within Carabidae and conducted phylogenetic analyses with broader taxon sampling across 21 subfamilies [40,42,43]. Results revealed that some subfamilies showed inconsistent topologies between the ML and BI trees based on four different datasets (PCGsAA, PCGsNT, PCGs123R, and PCGs12R) (Figures S6–S12). The topology obtained from the PCGs123R dataset is shown here (Figure 5).

In all phylogenetic trees, the monophyly of Cicindelinae, Elaphrinae, and Rhysodinae, and Harpalinae sensu stricto (Harpalinae s. str.) was highly supported (PP = 1, BS = 100). Cicindelinae exhibited two alternative topological patterns across different trees: it was either nested within Carabidae (PCGsNT and PCGsAA) or recovered as the basal-most lineage of the tree (PCGs123R and PCGs12R). Nebriinae and Carabinae, located at the base of the Carabidae phylogenetic tree, were recovered as monophyletic in all phylogenetic trees except those based on the PCGsAA dataset, in which *Notiophilus quadripunctatus* (Nebriinae) and *Cychrus caraboides* (Carabinae) formed a single clade that was sister to the remaining members of Carabinae. Pterostichinae was also recovered as a monophyletic group, with high bootstrap support values (BS = 93) in the ML tree based on PCGs12R dataset and high posterior probability (PP ≥ 0.91) in all BI trees, sometimes with low bootstrap in remaining ML trees.

In this study, Harpalinae sensu lato (Harpalinae s. l.) was defined as a large clade that includes Lebiinae, Ctenodactylinae, Licininae, Panagaeinae, Platyninae, Harpalinae s. str., and Pterostichinae. This clade was consistently recovered as monophyletic, similar to Pterostichinae. Within Harpalinae s. l., Platyninae was typically positioned toward the terminal branches of the phylogenetic trees and was recovered as polyphyletic in most analyses. Even in the few analyses in which it was recovered as monophyletic (PCGsAA and PCGs123R), nodal support values remained low. In all phylogenetic trees, Licininae exhibited a paraphyletic pattern across most nodes, with high bootstrap support values (BS ≥ 79) in ML trees and strong posterior probability support (PP ≥ 0.97) in BI trees for most relevant nodes, and *Panagaeinae* was consistently nested within the Licininae clade.

Trechinae was recovered as paraphyletic in both ML and BI trees across all datasets. It always constituted a clade with *Mastax latefasciata* (Brachininae) and Rhysodinae, and sometimes also included Omophroninae and Loricerinae. The only exception was observed in the BI tree based on the PCGs12R dataset, where Trechinae formed a clade exclusively with Rhysodinae, while Brachininae was recovered as monophyletic out of that clade with strong posterior probability support (PP = 0.96). In all other phylogenetic tree analyses, Brachininae was consistently polyphyletic. The monophyly of Lebiinae was strongly supported in the BI trees (PCGsNT, PCGs123R, and PCGs12R) (PP = 0.96), and received high support in the ML trees based on the same datasets (BS ≥ 83). In the PCGsAA analyses, Lebiinae was recovered as non-monophyletic, although this topology may reflect differences in phylogenetic signal among datasets.

For the eight subfamilies—Broscinae, Paussinae, Loricerinae, Dryptinae, Ctenodactylinae, Panagaeinae, Promecognathinae, and Omophroninae—only a single mitogenome was available for each subfamily in this study. Therefore, the monophyly of these subfamilies could not be reliably assessed due to insufficient taxon sampling. This limited taxon sampling represents a limitation of the present study, and additional mitochondrial genomic data will be required to further evaluate their phylogenetic relationships. In addition, several subfamily-level relationships showed instability among different datasets, suggesting that phylogenetic signals provided by mitochondrial datasets may be insufficient for resolving some relationships within Carabidae. For example, the phylogenetic placement of Dryptinae exhibited two different topological patterns: either it first formed a clade with *Brachinus* (Brachininae) that was subsequently sister to Harpalinae s. l. (PCGsNT and PCGsAA), or it formed together with Brachininae (excluding *Mastax*) and Harpalinae s. l. (PCGs123R and PCGs12R), a three-lineage relationship expressed as Brachininae (excl. *Mastax*) + (Dryptinae + Harpalinae s. l.).

## 4. Discussion

### 4.1. Mitochondrial Genome Characterization

In this study, the newly sequenced mitogenomes of *Poecilus fortipes*, *Poecilus gebleri*, *Carabus brandti*, *Pristosia nitidula*, and *Cephalosdrophus marinae* all exhibited the typical double-stranded, closed-circular structure and contained 37 canonical genes, similar to other complete mitogenomes of carabid beetles [63,64]. Furthermore, these newly sequenced mitochondrial genomes showed gene arrangements and genome organizations resembling those of typical coleopteran mitogenomes [65,66,67]. The total mitochondrial genome length is often affected by the length of the control region in all five carabid species. This observation is consistent with previous studies showing that variation in mitogenome size is primarily associated with differences in control region length in eukaryotes. Such variation in mitochondrial control region length may be associated with differences in the numbers of tandem repeats, which are frequently observed in Carabidae mitochondrial genomes and could significantly contribute to differences in mitogenome size [63,68]. There is a general preference for A and T bases over G and C bases in insect mitochondrial genomes [30,69]. Most PCGs used the conventional ATN (ATA/T/C/G), except for the *ND1* gene, which starts with the unconventional TTG. This finding is consistent with previous observations reported by Zhu et al. [43]. In the five carabid species, codon usage patterns and nucleotide composition biases were consistent with the overall A+T-rich nature of insect mitochondrial genomes. This AT bias, a hallmark feature of mitochondrial genome evolution, is generally attributed to multiple factors [70,71,72], including mutational pressures favoring A/T substitution [73], relaxed constraints on mitochondrial gene codon usage [74], and potential selection pressures related to DNA replication or transcription efficiency [75,76].

*ATP8* exhibited the highest Ka/Ks ratio among the PCGs of the five carabid species, whereas *COX1* shows the lowest. This indicates that *ATP8* experiences the weakest selective pressure and is the fastest-evolving gene among the mitochondrial PCGs of the five species analyzed. In contrast, *COX1* is under strong purifying selection, displaying the lowest evolutionary rate, high sequence conservation, and minimal genetic variability. This is a universal pattern in the evolution of insect mitochondrial genomes [77]. These results further highlight the heterogeneous evolutionary dynamics among mitochondrial genes. The high level of sequence conservation in *COX1* makes it a reliable molecular marker for species identification and phylogenetic reconstruction within Carabidae [78,79].

The secondary structures of the mitochondrial tRNAs in these five carabid beetles generally adopt the typical cloverleaf structure, with two exceptions: the DHU arm forms only a loop in tRNA-*Ser* (AGN), and the TΨC loop is missing in tRNA-*Phe* of *Cephalosdrophus marinae*. These structural anomalies appear to represent structural variations rather than phylogenetically conserved features, as similar mitochondrial tRNA modifications have been reported in several previously described metazoan mitochondrial genomes [80,81]. The DHU stem and loop are involved in tertiary interactions and play an important role in the proper folding and functioning of tRNA [82]. U-G mismatches were detected in the tRNA secondary structures of all five species, which also occur in other insect mitochondrial genomes [83,84]. Notably, the U-G mismatch is the most frequently encountered non-canonical base pair and is critical for the higher-order structure and function of RNA. Phylogenetically conserved U-G wobble pairs occur in most classes of structured RNAs [85].

### 4.2. Phylogenetic Analyses of Carabidae

The monophyly of Cicindelinae was strongly supported by ML and BI analyses based on four different datasets. In this study, however, the phylogenetic placement of Cicindelinae varied among mitochondrial datasets: it was nested within Carabidae in the PCGsNT and PCGsAA analyses, whereas it was positioned as the most basal lineage in the PCGs123R and PCGs12R analyses. These conflicting topologies suggest that different mitochondrial datasets contain heterogeneous phylogenetic signals. The topology recovered from the PCGs123R and PCGs12R datasets, namely Trachypachidae + (Cicindelinae + Carabidae), is consistent with previous transcriptomic, genomic, and morphological studies [28,86,87]. Nevertheless, the conflicting phylogenetic placements recovered from different mitochondrial datasets suggest that the phylogenetic position of *Cicindelinae* remains unresolved. Further studies incorporating independent nuclear genomic markers, such as nuclear genes and ultraconserved elements (UCEs), broader taxon sampling, and complementary phylogenomic datasets, will be necessary to further resolve the phylogenetic position of Cicindelinae. Here, both Nebriinae and Carabinae were recovered as monophyletic in most phylogenetic trees (except those based on the PCGsAA dataset) and occupied basal positions within the Carabidae phylogeny. This result is consistent with the majority of previous studies [43,86,88].

In agreement with earlier studies, the present phylogenetic analysis suggests that Harpalinae s. l. was consistently resolved as the sister group of Brachininae or Dryptinae, and that the latter two subfamilies together constitute the sister lineage to Trechinae [89,90,91]. Trechinae is the most species-rich group within Carabidae after Harpalinae s. l., comprising over 6700 species. It primarily includes two supertribes: Trechitae and Patrobitae. According to the Catalogue of Life (COL), Trechinae is currently divided into six tribes: Pogonini, Bembidiini, Zolini, Sinozolini, Bembidarenini, and Trechini. Because the majority of Trechinae species belong to the supertribe Trechitae, comprising over 5300 species [92], most previous studies have focused on the phylogenetic relationships of lineages within Trechitae, based on morphological data from adults and larvae of a limited number of taxa, as well as molecular data from a few gene sequences [93,94,95]. In our study, Trechinae was consistently recovered as paraphyletic (except in the PCGs12R-BI tree) and always formed a clade comprising *Mastax* (Brachininae) and Rhysodinae. No synapomorphies were shared between Trechinae and Rhysodinae. Although Trechinae and Brachininae exhibit some overlap in morphological characters and ecological habits—namely small body size and ground-dwelling habits—they can be distinguished by differences in aedeagus structure. Moreover, Brachininae lack the extreme troglobiomorphic specializations observed in the cave lineages of Trechinae. The observed phylogenetic pattern may be influenced by the limited taxonomic sampling of molecular data in this analysis, which included only six species representing three tribes (Bembidiini, Trechini, and Pogonini) within Trechinae. Therefore, the branching topology of Trechinae requires further investigation with more extensive sampling to be resolved.

Based on previous phylogenomic and morphological analyses, it is widely accepted that Rhysodinae belongs to Carabidae [86,96,97]. The results of this study also show that Rhysodinae is nested within Carabidae and strongly support its monophyly. Moreover, this clade exhibits a long-branch phenomenon in the phylogenetic tree, a finding consistent with earlier studies [42,43]. Such long-branch characteristics may increase the susceptibility to long-branch attraction, particularly in mitochondrial phylogenetic analyses where limited phylogenetic signals and compositional biases may affect topology reconstruction. Therefore, the unstable placement of Rhysodinae reported in some previous studies may result from a combination of mitochondrial evolutionary history and analytical limitations. However, when analyzing phylogenetic signals from mitochondrial genes, nuclear-encoded metazoan oxidative phosphorylation (OXPHOS) genes, and other nuclear genes in 53 beetle species, Zhao et al. [98] found evidence that the mitochondrial genomes of rhysodines have undergone introgressive hybridization events; these results support a phylogenetic placement of Rhysodinae outside Carabidae. This “loose mitonuclear interaction mechanism” needs further validation in future higher-level phylogenetic studies. Furthermore, in our results, Brachininae was consistently polyphyletic, except in the PCGs12R-BI tree, where it was recovered as monophyletic with a nodal support of 0.96. Numerous molecular studies have also favored the non-monophyly of this subfamily [26,42,89]. Morphologically, however, Brachininae possesses a unique feature, namely eight visible ventrites in males and seven in females instead of the typical six, which has been used to support its monophyly [43]. This conflicting pattern further suggests that the observed results may be due to insufficient phylogenetic signal from the mitochondrial genomes themselves. Interestingly, a close relationship between Brachininae and Dryptinae was recovered in our study, a finding recently confirmed by Garner et al. [91]. Additionally, in most topologies, Omophroninae and Loricerinae formed a small clade as sister groups. This relationship is consistent with the results of Vasilikopoulos et al. [86] and the study by Cardenas et al. [87], albeit with low nodal support. Both subfamilies are unusual in that each contains only a single genus, and in this study, a single representative species was available, similar to other subfamilies (Broscinae, Paussinae, Dryptinae, Ctenodactylinae, Panagaeinae, and Promecognathinae). Future studies should include increased taxon sampling within each subfamily to better resolve their phylogenetic positions within Carabidae and the relationships among them.

Within Harpalinae s. l., the monophyly of all constituent taxa is strongly supported except for Licininae and Platyninae. Notably, Pterostichinae occasionally exhibits low nodal support for its monophyly. This finding is consistent with recent mitogenome-based studies. Morphologically, however, Pterostichinae is a complex group comprising 231 genera placed in seven tribes, according to the COL database. This lineage lacks clear synapomorphies that would robustly support its monophyly; rather, the subfamily has traditionally been defined by a combination of morphological characters, including a relatively robust body and the presence of two setiferous punctures beside each eye [43,91]. The persistent instability in the phylogenetic placement of Pterostichinae is likely attributable to its high species diversity. In this study, molecular data for Pterostichinae were very limited, with sampling covering only seven genera belonging to two tribes. Although we newly sequenced two *Poecilus* species, thereby supplementing the molecular data for Pterostichini, this remains insufficient to robustly resolve the phylogenetic relationships within this group. Platyninae was the only subfamily within Harpalinae s. l. recovered as potentially polyphyletic, consistent with the findings of Zhu et al. [43]. Even in the few phylogenetic trees where it appeared as monophyletic, nodal support values remained low. These results suggest that mitochondrial genes may provide insufficient phylogenetic signals for resolving relationships within this group. In contrast, nuclear genomic data provide a larger number of independent loci and incorporate biparental inheritance signals, thereby offering a more comprehensive representation of species evolutionary histories. Therefore, future studies should incorporate nuclear genomic data to further resolve the phylogenetic relationships of Platyninae. This subfamily is divided into three tribes: Omphreini, Sphodrini, and Platynini. In our results, the monophyly of Sphodrini and Platynini was strongly supported, except in the phylogenetic trees based on the PCGsAA and PCGs123R datasets, where they clustered together with low nodal support. *Pristosia nitidula* and *Cephalosdrophus marinae* both belong to the tribe Sphodrini. The former represents the first mitochondrial genome sequence for the subtribe Pristosiina, while the latter represents the first mitochondrial genome sequence for the *Sphodrus* clade within the subtribe Sphodrina [99]. This study marks the first mitochondrial phylogenomic research encompassing all six subtribes of the tribe Sphodrini. The results show that the tribe Sphodrini and all six subtribes are monophyletic, with the phylogenetic relationships as follows: Atranopsina + ((Pristosiina + (Calathina + Dolichina)) + (Synuchina + Sphodrina)). All nodes are supported with 100. The basal position of the subtribe Atranopsina is consistent with previous conclusions based on multiple gene fragments, while the phylogenetic relationships among the remaining five subtribes are resolved for the first time [100]. Future research should focus on increasing sampling within the subtribe Dolichina, which includes many genera, as well as addressing the lack of data on the complex and large genus *Pseudotaphoxenus*.

## 5. Conclusions

In this study, the complete mitogenomes of *Poecilus fortipes*, *Poecilus gebleri*, *Pristosia nitidula* and *Cephalosdrophus marinae* were reported here for the first time, while the mitogenome of *Carabus brandti* was resequenced. The composition and length of the genes of these new mitogenomes are consistent with the organization of typical insect mitogenomes, and the selection pressure and evolutionary rates of each gene are consistent with those previously reported for carabids’ mitochondrial genes. Analyses of evolutionary rates indicated that *ATP8* and *COX1* exhibited the highest and lowest Ka/Ks values, respectively, suggesting substantial variation in selective constraints among mitochondrial genes.

Based on 124 mitochondrial genome sequences retrieved from NCBI, we expanded mitogenomic sampling for three genera within Carabidae (*Poecilus*, *Pristosia*, and *Cephalosdrophus*) and reconstructed the phylogenetic relationships of 21 subfamilies of Carabidae using four different datasets. Our results suggest that the phylogenetic position and taxonomic status of Cicindelinae remain unresolved, with alternative placements recovered depending on the dataset analyzed. The monophyly of most carabid subfamilies was supported by our analyses, with the exceptions of Brachininae, Trechinae, Licininae, and Platyninae. Nevertheless, many representative taxa remain either unsampled or underrepresented in current phylogenetic datasets, resulting in uncertainty regarding the placement of several lineages within Carabidae. Future studies should therefore prioritize expanding mitochondrial genome sampling in poorly represented groups. Furthermore, integrating broader genomic coverage, broader taxon sampling, and additional nuclear genomic markers (e.g., nuclear genes and UCEs) will be essential for achieving a more robust and comprehensive understanding of phylogenetic relationships within Carabidae.

## Figures and Tables

**Table 1:** The voucher information of the specimens sequenced in this study.

Number_ID	Species	Geographic Co.	Altitude	Date
GMEB_0016	*Poecilus fortipes* (Chaudoir, 1850)	105.90950° E, 38.77750° N	2280.4 m	July 2021
GMEB_0017	*Poecilus gebleri* (Dejean, 1828)	105.88485° E, 38.77945° N	2482.4 m	July 2021
GMEB_0018	*Carabus brandti* Faldermann, 1835	105.918661° E, 38.77235° N	2030.0 m	July 2022
GMEB_0019	*Pristosia nitidula* (A. Morawitz, 1862)	105.909550° E, 38.77750° N	2280.4 m	July 2021
GMEB_0020	*Cephalosdrophus marinae* (Lassalle & Marcilhac, 1999)	105.90060° E, 38.77800° N	2407.0 m	July 2021

## Data Availability

The newly sequenced mitochondrial genome sequences in this paper have been deposited in GenBank under accession numbers PX606622–PX606626.

## Supplementary Materials

The following supporting information can be downloaded at: https://www.mdpi.com/article/10.3390/biology15181593/s1, Figure S1: Secondary structure of tRNAs identified in the *Poecilus fortipes*; Figure S2: Secondary structure of tRNAs identified in the *Poecilus gebleri;* Figure S3: Secondary structure of tRNAs identified in the *Carabus brandti*; Figure S4: Secondary structure of tRNAs identified in the *Pristosia nitidula*; Figure S5: Secondary structure of tRNAs identified in the *Cephalosdrophus marinae*; Figure S6: The maximum likelihood tree based on PCGsAA dataset. The numbers near the nodes are bootstrap support values (BS); Figure S7: The Bayesian inference tree based on PCGsAA dataset. The numbers near the nodes are Bayesian posterior probabilities (PPs); Figure S8: The maximum likelihood tree based on PCGsNT dataset. The numbers near the nodes are bootstrap support values (BS); Figure S9: The Bayesian inference tree based on PCGsNT dataset. The numbers near the nodes are Bayesian posterior probabilities (PPs); Figure S10: The maximum likelihood tree based on PCGs12R dataset. The numbers near the nodes are bootstrap support values (BS); Figure S11: The Bayesian inference tree based on PCGs12R dataset. The numbers near the nodes are Bayesian posterior probabilities (PPs); Figure S12: The maximum likelihood tree based on PCGs123R dataset. The numbers near the nodes are bootstrap support values (BS); Table S1: List of carabid beetles analyzed in this study together with relevant information; Table S2: Annotation of the mitochondrial genomes of five carabid beetles; Table S3: RSCU of five carabid beetles; Table S4: The Ka, Ks, and Ka/Ks ratios of 13 PCGs among five carabid beetles; Table S5: The Base Data of the five carabid beetles.
