# Supplementary material for: Mitochondrial Genome Comparison and Phylogenetic Analysis of the Family Carabidae (Coleoptera: Adephaga)
Source: Biology (Basel). 2026 Sep 9;15(18):1593. doi: 10.3390/biology15181593 (PMC13604674; doi:10.3390/biology15181593)
Supplement: Supplementary file 1 [file biology-15-01593-s001.zip › biology-4526294-supplementary.pdf]

**Table S1:** List of carabid beetles analyzed in this study together with relevant information

| GenBank No. | Subfamily/Family | Tribe | Species | Length | Reference |
|---|---|---|---|---|---|
| JX313665.1 | Promecognathinae | Promecognathini | *Promecognathus crassus* | 16,535 bp | Timmermans et al, 2015 |
| JX412738.1 | Panagaeinae | Panagaeini | *Craspedophorus nobilis* | 15,063 bp | Timmermans et al, 2015 |
| JX412824.1 | Cicindelinae | Cicindelini | *Habrodera capensis* | 16,367 bp | Direct Submission |
| JX412826.1 | Brachininae | Brachinini | *Brachinus crepitans* | 14,457 bp | Direct Submission |
| JX412768.1 | Ctenodactylinae | Hexagoniini | *Hexagonia terminalis* | 12,639 bp | Direct Submission |
| KT876906.1 | Nebriinae | Nebriini | *Nebria brevicollis* | 21,161 bp | Linard et al, 2016 |
| KT876910.1 | Pterostichinae | Pterostichini | *Pterostichus madidus* | 18,324 bp | Linard et al, 2016 |
| KX035156.1 | Rhysodinae | Rhysodini | *Rhysodes* sp. | 16,315 bp | Direct Submission |
| KX087242.1 | Trechinae | Bembidiini | *Bembidion varium* | 16,224 bp | Direct Submission |
| KX087243.1 | Elaphrinae | Elaphrini | *Blethisa multipunctata* | 16,332 bp | Direct Submission |
| KX087286.1 | Elaphrinae | Elaphrini | *Elaphrus cupreus* | 16,317 bp | Direct Submission |
| KX087304.1 | Lebiinae | Lebiini | *Lebia chlorocephala* | 16,406 bp | Direct Submission |
| KX087338.1 | Trechinae | Pogonini | *Pogonus iridipennis* | 15,916 bp | Direct Submission |
| KX087349.1 | Pterostichinae | Pterostichini | *Stomis pumicatus* | 17,263 bp | Direct Submission |
| MF497813.1 | Cicindelinae | Platychilini | *Omus cazieri* | 18,150 bp | López-López & Vogler 2017 |
| MF497814.1 | Cicindelinae | Platychilini | *Platychile pallida* | 15,545 bp | López-López & Vogler 2017 |
| MF497816.1 | Cicindelinae | Megacephalini | *Australicapitona hopei* | 16,191 bp | López-López & Vogler 2017 |
| MF497817.1 | Paussinae | Metriini | *Metrius contractus* | 16,459 bp | López-López & Vogler 2017 |
| MF497818.1 | Cicindelinae | Cicindelini | *Odontocheila batesii* | 15,874 bp | López-López & Vogler 2017 |
| MF497819.1 | Broscinae | Broscini | *Broscus cephalotes* | 14,129 bp | López-López & Vogler 2017 |
| MF497820.1 | Cicindelinae | Collyridini | *Pogonostoma subtiligrossum* | 16,764 bp | López-López & Vogler 2017 |
| MF497822.1 | Scaritinae | Scaritini | *Scarites buparius* | 16,082 bp | López-López & Vogler 2017 |
| MG253281.1 | Cicindelinae | —— | Cicindelinae sp. | 20,282 bp | Direct Submission |
| MH789731.1 | Lebiinae | Lebiini | Lebiini sp. | 16,119 bp | Crampton-Platt et al, 2015 |
| MK692555.1 | Pterostichinae | Pterostichini | *Orthomus* sp. | 16,368 bp | Andujar et al, 2019 |
| MN122833.1 | Pterostichinae | Pterostichini | *Pterostichus oblongopunctatus* | 16,740 bp | Direct Submission |
| MN122835.1 | Carabinae | Carabini | *Carabus arvensis* | 16,892 bp | Direct Submission |
| MN122850.1 | Carabinae | Carabini | *Carabus hortensis* | 16,402 bp | Direct Submission |
| MN335930.1 | Pterostichinae | Zabrini | *Amara* sp. | 16,646 bp | Li et al, 2020 |
| MN480425.1 | Carabinae | Carabini | *Carabus smaragdinus* | 16,737 bp | Oh et al, 2020 |
| MT554376.1 | Licininae | Chlaeniini | *Chlaenius micans* | 17,053 bp | Direct Submission |
| MT554378.1 | Platyninae | Platynini | *Metacolpodes buchanani* | 16,561 bp | Direct Submission |
| MT554379.1 | Cicindelinae | Cicindelini | *Cicindela setosomalaris* | 17,378 bp | Direct Submission |
| MT554380.1 | Cicindelinae | Collyridini | *Tricondyla gestroi* | 16,146 bp | Direct Submission |
| MT872694.1 | Pterostichinae | Pterostichini | *Pterostichus melanarius* | 18,375 bp | Direct Submission |
| MT872702.1 | Loricerinae | Loricerini | *Loricera pilicornis* | 16,585 bp | Direct Submission |
| MT872703.1 | Carabinae | Cychrini | *Cychrus caraboides* | 17,213 bp | Direct Submission |
| MW074920.1 | Cicindelinae | Cicindelini | *Habroscelimorpha dorsalis dorsalis* | 16,404 bp | Direct Submission |
| MW113736.1 | Cicindelinae | Cicindelini | *Habroscelimorpha dorsalis media* | 16,404 bp | Direct Submission |
| MW244066.1 | Nebriinae | Nebriini | *Nebria ingens riversi* | 15,808 bp | Weng et al, 2021 |
| MW442537.1 | Cicindelinae | Cicindelini | *Cicindela puritana* | 15,676 bp | Direct Submission |

| GenBank No. | Subfamily/Family | Tribe | Species | Length | Reference |
|---|---|---|---|---|---|
| MW450884.1 | Cicindelinae | Cicindelini | *Habroscelimorpha dorsalis venusta* | 16,409 bp | Direct Submission |
| MW450885.1 | Cicindelinae | Cicindelini | *Habroscelimorpha dorsalis saulcyi* | 16,409 bp | Direct Submission |
| NC_016469.1 | Carabinae | Carabini | *Damaster mirabilissimus mirabilissimus* | 16,823 bp | Wan et al, 2012 |
| NC_018339.1 | Carabinae | Carabini | *Calosoma sp.* | 16,462 bp | Song et al, 2010 |
| NC_030592.1 | Pterostichinae | Pterostichini | *Abax parallelepipedus* | 17,701 bp | Linard et al, 2016 |
| NC_035714.1 | Cicindelinae | Platychilini | *Manticora tibialis* | 16,439 bp | López-López & Vogler 2017 |
| NC_036261.1 | Trechinae | Bembidiini | *Tachyta nana* | 16,165 bp | Direct Submission |
| NC_036268.1 | Pterostichinae | Zabrini | *Amara communis* | 15,745 bp | Direct Submission |
| NC_036507.1 | Carabinae | Carabini | *Carabus lafossei* | 16,793 bp | Liu et al, 2018 |
| NC_038191.1 | Cicindelinae | Cicindelini | *Abroscelis anchoralis* | 16,388 bp | Wang et al, 2018 |
| NC_044759.1 | Carabinae | Carabini | *Carabus granulatus* | 16,918 bp | Direct Submission |
| NC_044760.1 | Pterostichinae | Pterostichini | *Pterostichus niger* | 17,162 bp | Direct Submission |
| NC_045094.1 | Harpalinae | Harpalini | *Anisodactylus punctatipennis* | 16,521 bp | Yu et al, 2019 |
| NC_046469.1 | Carabinae | Carabini | *Carabus changeonleei* | 16,831 bp | Wang et al, 2019 |
| NC_046953.1 | Harpalinae | Harpalini | *Harpalus pensylvanicus* | 16,434 bp | Kieran, 2020 |
| NC_060736.1 | Scaritinae | Scaritini | *Scarites subterraneus* | 16,163 bp | Kyndt E & Kyndt J, 2022 |
| NC_064368.1 | Nebriinae | Notiophilini | *Omophron limbatum* | 15,438 bp | Raupach et al, 2023 |
| NC_064369.1 | Nebriinae | Notiophilini | *Notiophilus quadripunctatus* | 15,312 bp | Raupach et al, 2023 |
| NC_065263.1 | Brachininae | Brachinini | *Mastax latefasciata* | 16,735 bp | Bai et al, 2022b |
| NC_066078.1 | Harpalinae | Harpalini | *Nipponoharpalus discrepans* | 16,027 bp | Lin et al, 2024 |
| NC_066080.1 | Harpalinae | Harpalini | *Harpalus* sp. | 16,972 bp | Lin et al, 2024 |
| NC_066084.1 | Dryptinae | Galeritini | *Brachinus* sp. | 16,137 bp | Bai et al, 2022a |
| NC_067049.1 | Pterostichinae | Zabrini | *Amara ovata* | 15,334 bp | Lin et al, 2024 |
| NC_067050.1 | Licininae | Licinini | *Diplocheila zeelandica* | 16,190 bp | Lin et al, 2024 |
| NC_067745.1 | Rhysodinae | Rhysodini | *Rhyzodiastes puetzi* | 16,793 bp | Direct Submission |
| NC_067792.1 | Rhysodinae | Rhysodini | *Omoglymmius wukong* | 15,378 bp | Direct Submission |
| NC_068073.1 | Cicindelinae | Cicindelini | *Calomera brevipilosa* | 16,840 bp | Direct Submission |
| NC_072153.1 | Platyninae | Platynini | *Dyscolus rotundiceps* | 16,573 bp | Murienne et al, 2022 |
| NC_072154.1 | Platyninae | Platynini | *Dyscolus oreas* | 16,572 bp | Murienne et al, 2022 |
| NC_072155.1 | Platyninae | Platynini | *Dyscolus oopteroides* | 16,581 bp | Murienne et al, 2022 |
| NC_072156.1 | Platyninae | Platynini | *Dyscolus lucifuga* | 16,578 bp | Murienne et al, 2022 |
| NC_072157.1 | Platyninae | Platynini | *Dyscolus arauzae* | 16,546 bp | Murienne et al, 2022 |
| NC_072158.1 | Platyninae | Platynini | *Dyscolus diopsis* | 17,474 bp | Murienne et al, 2022 |
| NC_072159.1 | Platyninae | Platynini | *Dyscolus orthomus* | 17,084 bp | Murienne et al, 2022 |
| NC_072160.1 | Platyninae | Platynini | *Dyscolus fusipalpis* | 16,572 bp | Murienne et al, 2022 |
| NC_072161.1 | Platyninae | Platynini | *Dyscolus pollens* | 16,566 bp | Murienne et al, 2022 |
| NC_072162.1 | Platyninae | Platynini | *Dyscolus funereus* | 17,463 bp | Murienne et al, 2022 |
| NC_072163.1 | Platyninae | Platynini | *Dyscolus montivagus* | 16,571 bp | Murienne et al, 2022 |
| NC_072164.1 | Platyninae | Platynini | *Dyscolus irriguus* | 16,563 bp | Murienne et al, 2022 |
| NC_085183.1 | Platyninae | Sphodrini | *Synuchus nitidus* | 16392 bp | Kim et al, 2024 |

| GenBank No. | Subfamily/Family | Tribe | Species | Length | Reference |
|---|---|---|---|---|---|
| NC_086655.1 | Licininae | Chlaeniini | *Chlaenius naeviger* | 16,594 bp | Choi et al, 2024 |
| NC_086719.1 | Trechinae | Bembidiini | *Bembidion laterale* | 15990 bp | Raupach et al, 2024 |
| OP161481.1 | Carabinae | Carabini | *Carabus* sp. | 16,546 bp | Lin et al, 2024 |
| OQ621646.1 | Carabinae | Carabini | *Carabus leechi yooni* | 16,825 bp | Kwon et al, 2023 |
| OQ716306.1 | Trechinae | Trechini | *Aepus gracilicornis* | 16,685 bp | Jiménez-García et al, 2023 |
| OQ716310.1 | Platyninae | Sphodrini | *Amaroschema gaudini* | 13,830 bp | Jiménez-García et al, 2023 |
| OQ716321.1 | Harpalinae | Stenolophini | *Bradycellus ventricosus* | 16,791 bp | Jiménez-García et al, 2023 |
| OQ716324.1 | Platyninae | Sphodrini | *Calathidius sp.* | 17,786 bp | Jiménez-García et al, 2023 |
| OQ716325.1 | Platyninae | Sphodrini | *Calathus gomerensis* | 16,764 bp | Jiménez-García et al, 2023 |
| OQ716336.1 | Dryptinae | Zuphiini | *Dicrodontus aptinoides* | 15,837 bp | Jiménez-García et al, 2023 |
| OQ716337.1 | Lebiinae | Lebiini | *Dromius angustus* | 17,432 bp | Jiménez-García et al, 2023 |
| OQ716340.1 | Pterostichinae | Pterostichini | *Eutrichopus gonzalezi* | 16,368 bp | Jiménez-García et al, 2023 |
| OQ716349.1 | Platyninae | Sphodrini | *Laemostenus complanatus* | 16,997 bp | Jiménez-García et al, 2023 |
| OQ716355.1 | Nebriinae | Nebriini | *Leistus nubivagus* | 15,123 bp | Jiménez-García et al, 2023 |
| OQ716368.1 | Platyninae | Platynini | *Olisthopus sp.* | 16,775 bp | Jiménez-García et al, 2023 |
| OQ716374.1 | Lebiinae | Lebiini | *Paradromius* sp. | 16,770 bp | Jiménez-García et al, 2023 |
| OQ716375.1 | Pterostichinae | Sphodrini | *Paraeutrichopus* sp. | 15,733 bp | Jiménez-García et al, 2023 |
| OQ716376.1 | Lebiinae | Lebiini | *Philorhizus* sp. | 16,522 bp | Jiménez-García et al, 2023 |
| OQ716397.1 | Trechinae | Bembidiini | *Typhlocharis* sp. | 14,925 bp | Jiménez-García et al, 2023 |
| OR536810.1 | Licininae | Chlaeniini | *Chlaenius tetragonoderus* | 16,419 bp | Li et al, 2024 |
| OW121814.1 | Nebriinae | Nebriini | *Leistus spinibarbis* | 15,821 bp | Direct Submission |
| OW964842.1 | Harpalinae | Harpalini | *Ophonus ardosiacus* | 16,612 bp | Direct Submission |
| OX063289.1 | Nebriinae | Nebriini | *Nebria salina* | 24,721 bp | Direct Submission |
| OX384048.1 | Platyninae | Platynini | *Agonum fuliginosum* | 19,272 bp | Direct Submission |
| OX596293.1 | Harpalinae | Harpalini | *Harpalus rufipes* | 17,371 bp | Direct Submission |
| OY720467.1 | Harpalinae | Harpalini | *Harpalus rubripes* | 16,481 bp | Direct Submission |
| OZ012734.1 | Pterostichinae | Pterostichini | *Poecilus cupreus* | 16,794 bp | Direct Submission |
| OZ014572.1 | Scaritinae | Clivinini | *Clivina fossor* | 16,475 bp | Direct Submission |
| PV167288.1 | Harpalinae | Harpalini | *Harpalus hauserianus* | 16,983 bp | Zhu et al., 2025 |
| PV167289.1 | Harpalinae | Harpalini | *Harpalus indicus* | 16,612 bp | Zhu et al., 2025 |
| PV167290.1 | Harpalinae | Harpalini | *Harpalus sinicus* | 16,573 bp | Zhu et al., 2025 |
| PV167291.1 | Harpalinae | Harpalini | *Harpalus tridens* | 16,066 bp | Zhu et al., 2025 |
| PV167292.1 | Harpalinae | Harpalini | *Harpalus pseudotinctulus* | 16,266 bp | Zhu et al., 2025 |
| PV167293.1 | Harpalinae | Harpalini | *Harpalus tinctulus* | 16,405 bp | Zhu et al., 2025 |
| PV288324.1 | Carabinae | Carabini | *Carabus davidis* | 16,624 bp | Direct Submission |
| PV339714.1 | Carabinae | Carabini | *Carabus brandti* | 16,796 bp | Direct Submission |
| PV762937.1 | Pterostichinae | Pterostichini | *Pterostichus interruptus* | 17,149 bp | Direct Submission |
| PV766247.1 | Pterostichinae | Pterostichini | *Pterostichus procax* | 16,288 bp | Direct Submission |
| PV766248.1 | Pterostichinae | Pterostichini | *Pterostichus adstrictus* | 16,684 bp | Direct Submission |
| PV766249.1 | Pterostichinae | Pterostichini | *Pterostichus orientalis* | 17,177 bp | Direct Submission |
| PX240909.1 | Cicindelinae | Cicindelini | *Cicindela aurulenta* | 16,388 bp | Direct Submission |
| PX578919.1 | Licininae | Chlaeniini | *Chlaenius circumdatus* | 16,669 bp | Direct Submission |
| PX591248.1 | Brachininae | Brachinini | *Pheropsophus javanus* | 16,752 bp | Direct Submission |

| GenBank No. | Subfamily/Family | Tribe | Species | Length | Reference |
|---|---|---|---|---|---|
| GMEB_0016 | Pterostichinae | Pterostichini | *Poecilus fortipes*★ | 16,847 bp | This study |
| GMEB_0017 | Pterostichinae | Pterostichini | *Poecilus gebleri*★ | 15,800 bp | This study |
| GMEB_0018 | Carabidae | Carabini | *Carabus brandti*★ | 16,795 bp | This study |
| GMEB_0019 | Platyninae | Spodrini | *Pristosia nitidula*★ | 17,352 bp | This study |
| GMEB_0020 | Platyninae | Spodrini | *Cephalosdrophus marinae*★ | 16,552 bp | This study |
| NC_054236.1 | Trachypachidae | | *Trachypachus holmbergi* | 15,722 bp | Sheffield et al, 2008 |

Note: ★ indicates species newly sequenced in this study. *Trachypachus holmbergi* (Genbank number: NC_054236.1) is the outgroup.

**Table S2:** Annotation of the mitochondrial genomes of five carabid beetles

| Gene | Speices | Position (bp) | Length (bp) | Direction | Intergenic Nucleotides | Start codon | Stop codon |
|---|---|---|---|---|---|---|---|
| tRNA-Ile | *Poecilus fortipes* | 1-67 | 67 | forward | 0 | | |
| | *Poecilus gebleri* | 1-67 | 67 | forward | 0 | | |
| | *Carabus brandti* | 1-67 | 67 | forward | 0 | | |
| | *Pristosia nitidula* | 1-65 | 65 | forward | 0 | | |
| | *Cephalosdrophus marinae* | 1-66 | 66 | forward | 0 | | |
| tRNA-Gln | *Poecilus fortipes* | 74-142 | 69 | reverse | 6 | | |
| | *Poecilus gebleri* | 74-142 | 69 | reverse | 6 | | |
| | *Carabus brandti* | 82-150 | 69 | reverse | 14 | | |
| | *Pristosia nitidula* | 70-138 | 69 | reverse | 4 | | |
| | *Cephalosdrophus marinae* | 71-139 | 69 | reverse | 4 | | |
| tRNA-Met | *Poecilus fortipes* | 142-211 | 70 | forward | -1 | | |
| | *Poecilus gebleri* | 142-211 | 70 | forward | -1 | | |
| | *Carabus brandti* | 169-237 | 69 | forward | 18 | | |
| | *Pristosia nitidula* | 138-208 | 71 | forward | -1 | | |
| | *Cephalosdrophus marinae* | 164-233 | 70 | forward | 24 | | |
| ND2 CDS | *Poecilus fortipes* | 212-1240 | 1029 | forward | 0 | ATA | TAA |
| | *Poecilus gebleri* | 212-1222 | 1011 | forward | 0 | ATA | TAA |
| | *Carabus brandti* | 238-1266 | 1029 | forward | 0 | ATG | TAA |
| | *Pristosia nitidula* | 209-1239 | 1029 | forward | 0 | ATA | TAA |
| | *Cephalosdrophus marinae* | 234-1262 | 1029 | forward | 0 | ATA | TAA |
| tRNA-Trp | *Poecilus fortipes* | 1241-1311 | 71 | forward | 0 | | |
| | *Poecilus gebleri* | 1226-1296 | 71 | forward | 3 | | |
| | *Carabus brandti* | 1268-1335 | 68 | forward | 1 | | |
| | *Pristosia nitidula* | 1238-1308 | 71 | forward | 2 | | |
| | *Cephalosdrophus marinae* | 1262-1334 | 73 | forward | 1 | | |
| tRNA-Cys | *Poecilus fortipes* | 1345-1410 | 66 | reverse | 33 | | |
| | *Poecilus gebleri* | 1330-1395 | 66 | reverse | 33 | | |
| | *Carabus brandti* | 1513-1579 | 67 | reverse | 177 | | |
| | *Pristosia nitidula* | 1342-1407 | 66 | reverse | 33 | | |
| | *Cephalosdrophus marinae* | 1363-1422 | 60 | reverse | 28 | | |

| Gene | Speices | Position (bp) | Length (bp) | Direction | Intergenic Nucleotides | Start codon | Stop codon |
|---|---|---|---|---|---|---|---|
| tRNA-Tyr | *Poecilus fortipes* | 1411-1478 | 68 | reverse | 0 | | |
| | *Poecilus gebleri* | 1396-1463 | 68 | reverse | 0 | | |
| | *Carabus brandti* | 1595-1661 | 67 | reverse | 15 | | |
| | *Pristosia nitidula* | 1533-1598 | 66 | reverse | 125 | | |
| | *Cephalosdrophus marinae* | 1602-1668 | 67 | reverse | 179 | | |
| COX1 CDS | *Poecilus fortipes* | 1471-3004 | 1534 | forward | -8 | ATT | T |
| | *Poecilus gebleri* | 1456-2989 | 1534 | forward | -8 | ATT | T |
| | *Carabus brandti* | 1654-3193 | 1540 | forward | -8 | ATT | T |
| | *Pristosia nitidula* | 1591-3130 | 1540 | forward | -8 | ATT | T |
| | *Cephalosdrophus marinae* | 1661-3200 | 1540 | forward | -8 | ATT | T |
| tRNA-Leu2 | *Poecilus fortipes* | 3011-3076 | 66 | forward | 6 | | |
| | *Poecilus gebleri* | 2996-3061 | 66 | forward | 6 | | |
| | *Carabus brandti* | 3194-3260 | 67 | forward | 0 | | |
| | *Pristosia nitidula* | 3131-3195 | 65 | forward | 0 | | |
| | *Cephalosdrophus marinae* | 3201-3265 | 65 | forward | 0 | | |
| COX2 CDS | *Poecilus fortipes* | 3078-3065 | 688 | forward | 1 | ATG | T |
| | *Poecilus gebleri* | 3063-3750 | 688 | forward | 1 | ATG | T |
| | *Carabus brandti* | 3262-3949 | 688 | forward | 1 | ATG | T |
| | *Pristosia nitidula* | 3197-3884 | 688 | forward | 1 | ATG | T |
| | *Cephalosdrophus marinae* | 3267-3954 | 688 | forward | 1 | ATG | T |
| tRNA-Lys | *Poecilus fortipes* | 3766-3836 | 71 | forward | 0 | | |
| | *Poecilus gebleri* | 3751-3821 | 71 | forward | 0 | | |
| | *Carabus brandti* | 3950-4020 | 71 | forward | 0 | | |
| | *Pristosia nitidula* | 3885-3955 | 71 | forward | 0 | | |
| | *Cephalosdrophus marinae* | 3955-4025 | 71 | forward | 0 | | |
| tRNA-Asp | *Poecilus fortipes* | 3838-3905 | 68 | forward | 1 | | |
| | *Poecilus gebleri* | 3823-3890 | 68 | forward | 1 | | |
| | *Carabus brandti* | 4027-4092 | 66 | forward | 6 | | |
| | *Pristosia nitidula* | 3956-4020 | 65 | forward | 0 | | |
| | *Cephalosdrophus marinae* | 4026-4091 | 66 | forward | | | |
| ATP8 CDS | *Poecilus fortipes* | 3906-4070 | 165 | forward | 0 | ATC | TAA |
| | *Poecilus gebleri* | 3891-4055 | 165 | forward | 0 | ATT | TAA |
| | *Carabus brandti* | 4093-4257 | 165 | forward | 0 | ATT | TAA |
| | *Pristosia nitidula* | 4021-4185 | 165 | forward | 0 | ATT | TAA |
| | *Cephalosdrophus marinae* | 4092-4253 | 162 | forward | 0 | ATT | TAA |
| ATP6 CDS | *Poecilus fortipes* | 4067-4741 | 675 | forward | -4 | ATA | TAA |
| | *Poecilus gebleri* | 4052-4726 | 675 | forward | -4 | ATA | TAA |
| | *Carabus brandti* | 4251-4928 | 678 | forward | -7 | ATG | TAA |
| | *Pristosia nitidula* | 4179-4856 | 678 | forward | -7 | ATG | TAA |
| | *Cephalosdrophus marinae* | 4247-4924 | 678 | forward | -7 | ATG | TAA |

| Gene | Speices | Position (bp) | Length (bp) | Direction | Intergenic Nucleotides | Start codon | Stop codon |
|---|---|---|---|---|---|---|---|
| COX3 CDS | *Poecilus fortipes* | 4752-5540 | 789 | forward | 10 | ATG | TAA |
| | *Poecilus gebleri* | 4737-5525 | 789 | forward | 10 | ATG | TAA |
| | *Carabus brandti* | 4928-5716 | 789 | forward | -1 | ATG | TAA |
| | *Pristosia nitidula* | 4863-5651 | 789 | forward | 6 | ATG | TAA |
| | *Cephalosdrophus marinae* | 4935-5723 | 789 | forward | 10 | ATG | TAA |
| tRNA-Gly | *Poecilus fortipes* | 5551-5616 | 66 | forward | 10 | | |
| | *Poecilus gebleri* | 5536-5601 | 66 | forward | 10 | | |
| | *Carabus brandti* | 5720-5785 | 66 | forward | 3 | | |
| | *Pristosia nitidula* | 5657-5722 | 66 | forward | 5 | | |
| | *Cephalosdrophus marinae* | 5729-5794 | 66 | forward | 5 | | |
| ND3 CDS | *Poecilus fortipes* | 5617-5970 | 354 | forward | 0 | ATT | TAG |
| | *Poecilus gebleri* | 5602-5955 | 354 | forward | 0 | ATT | TAA |
| | *Carabus brandti* | 5786-6139 | 354 | forward | 0 | ATT | TAG |
| | *Pristosia nitidula* | 5723-6076 | 354 | forward | 0 | ATT | TAA |
| | *Cephalosdrophus marinae* | 5795-6148 | 354 | forward | 0 | ATC | TAG |
| tRNA-Ala | *Poecilus fortipes* | 5969-6033 | 65 | forward | -2 | | |
| | *Poecilus gebleri* | 5955-6019 | 65 | forward | -1 | | |
| | *Carabus brandti* | 6138-6204 | 67 | forward | -2 | | |
| | *Pristosia nitidula* | 6076-6139 | 64 | forward | -1 | | |
| | *Cephalosdrophus marinae* | 6147-6212 | 66 | forward | -2 | | |
| tRNA-Arg | *Poecilus fortipes* | 6034-6101 | 65 | forward | 0 | | |
| | *Poecilus gebleri* | 6020-6087 | 68 | forward | 0 | | |
| | *Carabus brandti* | 6220-6284 | 65 | forward | 15 | | |
| | *Pristosia nitidula* | 6140-6204 | 65 | forward | 0 | | |
| | *Cephalosdrophus marinae* | 6216-6282 | 67 | forward | 3 | | |
| tRNA-Asn | *Poecilus fortipes* | 6104-6168 | 65 | forward | 2 | | |
| | *Poecilus gebleri* | 6090-6154 | 65 | forward | 2 | | |
| | *Carabus brandti* | 6289-6353 | 65 | forward | 4 | | |
| | *Pristosia nitidula* | 6208-6272 | 65 | forward | 3 | | |
| | *Cephalosdrophus marinae* | 6285-6349 | 65 | forward | 2 | | |
| tRNA-Ser1 | *Poecilus fortipes* | 6169-6235 | 67 | forward | 0 | | |
| | *Poecilus gebleri* | 6155-6221 | 67 | forward | 0 | | |
| | *Carabus brandti* | 6354-6420 | 67 | forward | 0 | | |
| | *Pristosia nitidula* | 6273-6339 | 67 | forward | 0 | | |
| | *Cephalosdrophus marinae* | 6350-6416 | 67 | forward | 0 | | |
| tRNA-Glu | *Poecilus fortipes* | 6242-6308 | 67 | forward | 6 | | |
| | *Poecilus gebleri* | 6223-6289 | 67 | forward | 1 | | |
| | *Carabus brandti* | 6422-6488 | 67 | forward | 1 | | |
| | *Pristosia nitidula* | 6341-6408 | 68 | forward | 1 | | |
| | *Cephalosdrophus marinae* | 6418-6484 | 67 | forward | 1 | | |

| Gene | Speices | Position (bp) | Length (bp) | Direction | Intergenic Nucleotides | Start codon | Stop codon |
|---|---|---|---|---|---|---|---|
| tRNA-Phe | *Poecilus fortipes* | 6307-6374 | 68 | reverse | -2 | | |
| | *Poecilus gebleri* | 6288-6355 | 68 | reverse | -1 | | |
| | *Carabus brandti* | 6509-6575 | 67 | reverse | 20 | | |
| | *Pristosia nitidula* | 6407-6473 | 67 | reverse | -2 | | |
| | *Cephalosdrophus marinae* | 6513-6572 | 60 | reverse | 28 | | |
| ND5 CDS | *Poecilus fortipes* | 6375-8103 | 1729 | reverse | 0 | ATT | T |
| | *Poecilus gebleri* | 6356-8084 | 1729 | reverse | 0 | ATT | T |
| | *Carabus brandti* | 6583-8313 | 1731 | reverse | 7 | ATT | TAA |
| | *Pristosia nitidula* | 6474-8202 | 1729 | reverse | 0 | ATT | T |
| | *Cephalosdrophus marinae* | 6573-8301 | 1729 | reverse | 0 | ATT | T |
| tRNA-His | *Poecilus fortipes* | 8104-8172 | 69 | reverse | 0 | | |
| | *Poecilus gebleri* | 8085-8153 | 69 | reverse | 0 | | |
| | *Carabus brandti* | 8314-8381 | 68 | reverse | 0 | | |
| | *Pristosia nitidula* | 8203-8269 | 67 | reverse | 0 | | |
| | *Cephalosdrophus marinae* | 8302-8368 | 67 | reverse | 0 | | |
| ND4 CDS | *Poecilus fortipes* | 8175-9513 | 1339 | reverse | 2 | ATG | T |
| | *Poecilus gebleri* | 8156-9494 | 1339 | reverse | 2 | ATG | T |
| | *Carabus brandti* | 8384-9724 | 1341 | reverse | 2 | ATG | TAA |
| | *Pristosia nitidula* | 8271-9593 | 1323 | reverse | 1 | ATA | TAA |
| | *Cephalosdrophus marinae* | 8370-9710 | 1341 | reverse | 1 | ATG | TAA |
| ND4L CDS | *Poecilus fortipes* | 9507-9800 | 294 | reverse | -7 | ATT | TAA |
| | *Poecilus gebleri* | 9488-9781 | 294 | reverse | -7 | ATT | TAA |
| | *Carabus brandti* | 9718-10011 | 294 | reverse | -7 | ATT | TAA |
| | *Pristosia nitidula* | 9605-9898 | 294 | reverse | 11 | ATT | TAA |
| | *Cephalosdrophus marinae* | 9704-9997 | 294 | reverse | -7 | ATT | TAA |
| tRNA-Thr | *Poecilus fortipes* | 9803-9867 | 65 | forward | 2 | | |
| | *Poecilus gebleri* | 9784-9848 | 65 | forward | 2 | | |
| | *Carabus brandti* | 1014-10078 | 65 | forward | 2 | | |
| | *Pristosia nitidula* | 9901-9965 | 65 | forward | 2 | | |
| | *Cephalosdrophus marinae* | 10000-10064 | 65 | forward | 2 | | |
| tRNA-Pro | *Poecilus fortipes* | 9868-9933 | 66 | reverse | 0 | | |
| | *Poecilus gebleri* | 9849-9914 | 66 | reverse | 0 | | |
| | *Carabus brandti* | 10079-10144 | 66 | reverse | 0 | | |
| | *Pristosia nitidula* | 9966-10031 | 66 | reverse | 0 | | |
| | *Cephalosdrophus marinae* | 10065-10131 | 67 | reverse | 0 | | |
| ND6 CDS | *Poecilus fortipes* | 9935-10459 | 525 | forward | 1 | ATT | TAA |
| | *Poecilus gebleri* | 9916-10440 | 525 | forward | 1 | ATT | TAA |
| | *Carabus brandti* | 10147-10671 | 525 | forward | 1 | ATT | TAA |
| | *Pristosia nitidula* | 10033-10557 | 525 | forward | 1 | ATT | TAA |
| | *Cephalosdrophus marinae* | 10133-10657 | 525 | forward | 1 | ATT | TAA |

| Gene | Speices | Position (bp) | Length (bp) | Direction | Intergenic Nucleotides | Start codon | Stop codon |
|---|---|---|---|---|---|---|---|
| CYTB CDS | *Poecilus fortipes* | 10459-11598 | 1140 | forward | -1 | ATG | TAA |
| | *Poecilus gebleri* | 10440-11579 | 1140 | forward | -1 | ATG | TAA |
| | *Carabus brandti* | 10671-11807 | 1137 | forward | -1 | ATG | TAG |
| | *Pristosia nitidula* | 10557-11696 | 1140 | forward | -1 | ATG | TAA |
| | *Cephalosdrophus marinae* | 10657-11796 | 1140 | forward | -1 | ATG | TAG |
| tRNA-Ser2 | *Poecilus fortipes* | 11601-11668 | 68 | forward | 2 | | |
| | *Poecilus gebleri* | 11582-11649 | 68 | forward | 2 | | |
| | *Carabus brandti* | 11806-11873 | 68 | forward | -2 | | |
| | *Pristosia nitidula* | 11695-11761 | 67 | forward | -2 | | |
| | *Cephalosdrophus marinae* | 11795-11861 | 67 | forward | -2 | | |
| ND1 CDS | *Poecilus fortipes* | 11686-12636 | 951 | reverse | 17 | TTG | TAG |
| | *Poecilus gebleri* | 11667-12617 | 951 | reverse | 17 | TTG | TAG |
| | *Carabus brandti* | 11891-12841 | 951 | reverse | 17 | TTG | TAG |
| | *Pristosia nitidula* | 11778-12728 | 951 | reverse | 16 | TTG | TAG |
| | *Cephalosdrophus marinae* | 11878-12828 | 951 | reverse | 16 | TTG | TAG |
| tRNA-Leu1 | *Poecilus fortipes* | 12638-12702 | 65 | reverse | 1 | | |
| | *Poecilus gebleri* | 12649-12683 | 65 | reverse | 1 | | |
| | *Carabus brandti* | 12843-12906 | 64 | reverse | 1 | | |
| | *Pristosia nitidula* | 12730-12793 | 64 | reverse | 1 | | |
| | *Cephalosdrophus marinae* | 12830-12894 | 65 | reverse | 1 | | |
| 16S rRNA | *Poecilus fortipes* | 12703-14018 | 1316 | reverse | 0 | | |
| | *Poecilus gebleri* | 12684-14001 | 1318 | reverse | 0 | | |
| | *Carabus brandti* | 12907-14242 | 1336 | reverse | 6 | | |
| | *Pristosia nitidula* | 12798-14105 | 1309 | reverse | 4 | | |
| | *Cephalosdrophus marinae* | 12894-14206 | 1313 | reverse | -1 | | |
| tRNA-Val | *Poecilus fortipes* | 14019-4090 | 72 | reverse | 0 | | |
| | *Poecilus gebleri* | 14001-14072 | 72 | reverse | -1 | | |
| | *Carabus brandti* | 14243-14313 | 71 | reverse | 0 | | |
| | *Pristosia nitidula* | 14103-14173 | 71 | reverse | -4 | | |
| | *Cephalosdrophus marinae* | 14207-14277 | 71 | reverse | 0 | | |
| 12S rRNA | *Poecilus fortipes* | 14091-14876 | 786 | reverse | 0 | | |
| | *Poecilus gebleri* | 14073-14858 | 786 | reverse | 0 | | |
| | *Carabus brandti* | 14314-15104 | 791 | reverse | 0 | | |
| | *Pristosia nitidula* | 14174-14959 | 786 | reverse | 0 | | |
| | *Cephalosdrophus marinae* | 14278-15066 | 789 | reverse | 0 | | |
| A+T-rich region | *Poecilus fortipes* | 14877-16847 | 1971 | forward | 0 | | |
| | *Poecilus gebleri* | 14859-15800 | 942 | forward | 0 | | |
| | *Carabus brandti* | 15105-16795 | 1691 | forward | 0 | | |
| | *Pristosia nitidula* | 14960-17352 | 2393 | forward | 0 | | |
| | *Cephalosdrophus marinae* | 15067-16552 | 1486 | forward | 0 | | |

**Table S3:** RSCU of five carabid beetles

| Amino acid | Codon | *Poecilus fortipes* | | *Poecilus gebleri* | | *Carabus brandti* | | *Pristosia nitidula* | | *Cephalosdrophus marinae* | |
|---|---|---|---|---|---|---|---|---|---|---|---|
| | | Number | RSCU | Number | RSCU | Number | RSCU | Number | RSCU | Number | RSCU |
| Phe | UUU(F) | 329 | 1.9 | 324 | 1.87 | 324 | 1.84 | 331 | 1.88 | 316 | 1.76 |
| | UUC(F) | 18 | 0.1 | 23 | 0.13 | 29 | 0.16 | 21 | 0.12 | 43 | 0.24 |
| Leu | UUA(L) | 496 | 5.04 | 482 | 4.97 | 477 | 4.97 | 473 | 4.97 | 444 | 4.69 |
| | UUG(L) | 12 | 0.12 | 16 | 0.16 | 28 | 0.29 | 19 | 0.2 | 30 | 0.32 |
| | CUU(L) | 49 | 0.5 | 53 | 0.55 | 42 | 0.44 | 51 | 0.54 | 40 | 0.42 |
| | CUC(L) | 3 | 0.03 | 4 | 0.04 | 3 | 0.03 | 2 | 0.02 | 6 | 0.06 |
| | CUA(L) | 29 | 0.29 | 26 | 0.27 | 22 | 0.23 | 25 | 0.26 | 44 | 0.46 |
| | CUG(L) | 2 | 0.02 | 1 | 0.01 | 4 | 0.04 | 1 | 0.01 | 4 | 0.04 |
| Ile | AUU(I) | 341 | 1.92 | 336 | 1.91 | 355 | 1.88 | 354 | 1.85 | 354 | 1.82 |
| | AUC(I) | 14 | 0.08 | 16 | 0.09 | 22 | 0.12 | 28 | 0.15 | 35 | 0.18 |
| Met | AUA(M) | 268 | 1.91 | 258 | 1.81 | 286 | 1.88 | 280 | 1.89 | 273 | 1.83 |
| | AUG(M) | 13 | 0.09 | 27 | 0.19 | 19 | 0.12 | 16 | 0.11 | 25 | 0.17 |
| Val | GUU(V) | 80 | 1.74 | 84 | 1.78 | 60 | 1.52 | 66 | 1.62 | 64 | 1.67 |
| | GUC(V) | 0 | 0 | 0 | 0 | 4 | 0.1 | 2 | 0.05 | 6 | 0.16 |
| | GUA(V) | 102 | 2.22 | 100 | 2.12 | 89 | 2.25 | 90 | 2.21 | 78 | 2.04 |
| | GUG(V) | 2 | 0.04 | 5 | 0.11 | 5 | 0.13 | 5 | 0.12 | 5 | 0.13 |
| Ser | UCU(S) | 92 | 2.16 | 94 | 2.22 | 115 | 2.77 | 99 | 2.34 | 105 | 2.39 |
| | UCC(S) | 7 | 0.16 | 8 | 0.19 | 11 | 0.27 | 4 | 0.09 | 15 | 0.34 |
| | UCA(S) | 118 | 2.78 | 114 | 2.69 | 81 | 1.95 | 117 | 2.77 | 111 | 2.52 |
| | UCG(S) | 2 | 0.05 | 4 | 0.09 | 3 | 0.07 | 0 | 0 | 1 | 0.02 |
| Pro | CCU(P) | 74 | 2.26 | 74 | 2.26 | 74 | 2.29 | 78 | 2.44 | 74 | 2.31 |
| | CCC(P) | 10 | 0.31 | 8 | 0.24 | 12 | 0.37 | 7 | 0.22 | 22 | 0.69 |
| | CCA(P) | 43 | 1.31 | 47 | 1.44 | 39 | 1.21 | 42 | 1.31 | 28 | 0.88 |
| | CCG(P) | 4 | 0.12 | 2 | 0.06 | 4 | 0.12 | 1 | 0.03 | 4 | 0.12 |
| Thr | ACU(T) | 86 | 1.93 | 83 | 1.89 | 95 | 2.09 | 88 | 2.02 | 77 | 1.79 |
| | ACC(T) | 7 | 0.16 | 7 | 0.16 | 14 | 0.31 | 6 | 0.14 | 8 | 0.19 |
| | ACA(T) | 85 | 1.91 | 86 | 1.95 | 72 | 1.58 | 79 | 1.82 | 84 | 1.95 |
| | ACG(T) | 0 | 0 | 0 | 0 | 1 | 0.02 | 1 | 0.02 | 3 | 0.07 |
| Ala | GCU(A) | 81 | 2.23 | 84 | 2.32 | 93 | 2.58 | 94 | 2.56 | 84 | 2.29 |
| | GCC(A) | 5 | 0.14 | 8 | 0.22 | 9 | 0.25 | 7 | 0.19 | 17 | 0.46 |
| | GCA(A) | 59 | 1.63 | 49 | 1.35 | 41 | 1.14 | 45 | 1.22 | 44 | 1.2 |
| | GCG(A) | 0 | 0 | 4 | 0.11 | 1 | 0.03 | 1 | 0.03 | 2 | 0.05 |
| Tyr | UAU(Y) | 173 | 1.86 | 172 | 1.88 | 164 | 1.83 | 172 | 1.87 | 164 | 1.87 |
| | UAC(Y) | 13 | 0.14 | 11 | 0.12 | 15 | 0.17 | 12 | 0.13 | 11 | 0.13 |
| His | CAU(H) | 65 | 1.86 | 63 | 1.8 | 64 | 1.78 | 69 | 1.94 | 62 | 1.7 |
| | CAC(H) | 5 | 0.14 | 7 | 0.2 | 8 | 0.22 | 2 | 0.06 | 11 | 0.3 |
| Gln | CAA(Q) | 67 | 1.89 | 67 | 1.89 | 64 | 1.88 | 68 | 1.92 | 61 | 1.77 |
| | CAG(Q) | 4 | 0.11 | 4 | 0.11 | 4 | 0.12 | 3 | 0.08 | 8 | 0.23 |

| Amino acid | Codon | *Poecilus fortipes* | | *Poecilus gebleri* | | *Carabus brandti* | | *Pristosia nitidula* | | *Cephalosdrophus marinae* | |
|---|---|---|---|---|---|---|---|---|---|---|---|
| | | Number | RSCU | Number | RSCU | Number | RSCU | Number | RSCU | Number | RSCU |
| Asn | AAU(N) | 196 | 1.89 | 194 | 1.87 | 193 | 1.82 | 201 | 1.91 | 191 | 1.86 |
| | AAC(N) | 11 | 0.11 | 14 | 0.13 | 19 | 0.18 | 9 | 0.09 | 14 | 0.14 |
| Lys | AAA(K) | 76 | 1.63 | 78 | 1.66 | 87 | 1.72 | 86 | 1.81 | 80 | 1.63 |
| | AAG(K) | 17 | 0.37 | 16 | 0.34 | 14 | 0.28 | 9 | 0.19 | 18 | 0.37 |
| Asp | GAU(D) | 62 | 1.8 | 61 | 1.77 | 67 | 1.94 | 68 | 1.92 | 63 | 1.88 |
| | GAC(D) | 7 | 0.2 | 8 | 0.23 | 2 | 0.06 | 3 | 0.08 | 4 | 0.12 |
| Glu | GAA(E) | 76 | 1.92 | 75 | 1.9 | 69 | 1.77 | 74 | 1.95 | 65 | 1.71 |
| | GAG(E) | 3 | 0.08 | 4 | 0.1 | 9 | 0.23 | 2 | 0.05 | 11 | 0.29 |
| Cys | UGU(C) | 38 | 2 | 36 | 1.89 | 37 | 2 | 35 | 1.94 | 35 | 1.94 |
| | UGC(C) | 0 | 0 | 2 | 0.11 | 0 | 0 | 1 | 0.06 | 1 | 0.06 |
| Trp | UGA(W) | 94 | 1.96 | 95 | 1.98 | 91 | 1.9 | 94 | 1.96 | 87 | 1.81 |
| | UGG(W) | 2 | 0.04 | 1 | 0.02 | 5 | 0.1 | 2 | 0.04 | 9 | 0.19 |
| Arg | CGU(R) | 12 | 0.86 | 11 | 0.79 | 19 | 1.36 | 12 | 0.86 | 11 | 0.77 |
| | CGC(R) | 0 | 0 | 0 | 0 | 2 | 0.14 | 0 | 0 | 0 | 0 |
| | CGA(R) | 44 | 3.14 | 45 | 3.21 | 33 | 2.36 | 43 | 3.07 | 39 | 2.74 |
| | CGG(R) | 0 | 0 | 0 | 0 | 2 | 0.14 | 1 | 0.07 | 7 | 0.49 |
| Ser | AGU(S) | 53 | 1.25 | 49 | 1.16 | 35 | 0.84 | 38 | 0.9 | 39 | 0.89 |
| | AGC(S) | 7 | 0.16 | 10 | 0.24 | 3 | 0.07 | 1 | 0.02 | 3 | 0.07 |
| | AGA(S) | 61 | 1.44 | 60 | 1.42 | 83 | 2 | 79 | 1.87 | 77 | 1.75 |
| | AGG(S) | 0 | 0 | 0 | 0 | 1 | 0.02 | 0 | 0 | 1 | 0.02 |
| Gly | GGU(G) | 51 | 0.97 | 45 | 0.85 | 46 | 0.9 | 51 | 0.99 | 41 | 0.78 |
| | GGC(G) | 2 | 0.04 | 3 | 0.06 | 1 | 0.02 | 3 | 0.06 | 7 | 0.13 |
| | GGA(G) | 135 | 2.57 | 132 | 2.5 | 135 | 2.63 | 140 | 2.71 | 134 | 2.54 |
| | GGG(G) | 22 | 0.42 | 31 | 0.59 | 23 | 0.45 | 13 | 0.25 | 29 | 0.55 |
| ※ | UAA(*) | 7 | 1.56 | 8 | 1.78 | 8 | 1.45 | 6 | 1.71 | 6 | 1.33 |
| | UAG(*) | 2 | 0.44 | 1 | 0.22 | 3 | 0.55 | 1 | 0.29 | 3 | 0.67 |
| | codons | 3736 | | 3730 | | 3740 | | 3731 | | 3738 | |

**Table S4:** The Ka, Ks, and Ka/Ks ratios of 13 PCGs among five carabid beetles

| Gene | ATP6 | ATP8 | COX1 | COX2 | COX3 | CYTB | ND1 | ND2 | ND3 | ND4 | ND4L | ND5 | ND6 |
|---|---|---|---|---|---|---|---|---|---|---|---|---|---|
| Ka | 0.041 | 0.133 | 0.026 | 0.047 | 0.053 | 0.055 | 0.033 | 0.102 | 0.066 | 0.038 | 0.064 | 0.055 | 0.133 |
| Ks | 0.552 | 0.334 | 0.572 | 0.526 | 0.595 | 0.540 | 0.411 | 0.439 | 0.584 | 0.368 | 0.292 | 0.393 | 0.533 |
| Ka/Ks | 0.074 | 0.398 | 0.045 | 0.089 | 0.089 | 0.101 | 0.080 | 0.232 | 0.113 | 0.102 | 0.218 | 0.141 | 0.250 |

**Table S5:** The Base Data of the five carabid beetles

| Species | Gene | A | T | C | G | A+T | C + G | AT-skew | GC-skew | All (bp) |
|---|---|---|---|---|---|---|---|---|---|---|
| *P. fortipes* | Whole genome | 6,916 | 6,514 | 1,972 | 1,445 | 13,430 | 3,417 | 0.030 | -0.154 | 16847 |
| | PCGs | 4,486 | 4,240 | 1,405 | 1,081 | 8,726 | 2,486 | 0.028 | -0.130 | 11212 |
| | tRNAs | 609 | 571 | 169 | 138 | 1,180 | 307 | 0.032 | -0.101 | 1487 |
| | rRNAs | 907 | 820 | 245 | 130 | 1,727 | 375 | 0.050 | -0.307 | 2102 |
| | A + T rich region | 879 | 850 | 149 | 93 | 1,729 | 242 | 0.017 | -0.231 | 1971 |
| *P. gebleri* | Whole genome | 6,421 | 5,955 | 1,965 | 1,459 | 12,376 | 3,424 | 0.038 | -0.148 | 15800 |
| | PCGs | 4,453 | 4,199 | 1,444 | 1,099 | 8,652 | 2,543 | 0.029 | -0.136 | 11195 |
| | tRNAs | 605 | 572 | 168 | 142 | 1,177 | 310 | 0.028 | -0.084 | 1487 |
| | rRNAs | 906 | 820 | 246 | 132 | 1,726 | 378 | 0.050 | -0.302 | 2104 |
| | A + T rich region | 419 | 338 | 102 | 83 | 757 | 185 | 0.107 | -0.103 | 942 |
| *C. brandti* | Whole genome | 6,903 | 6,471 | 2,012 | 1,409 | 13,374 | 3,421 | 0.032 | -0.176 | 16795 |
| | PCGs | 4,467 | 4,244 | 1,443 | 1,068 | 8,711 | 2,511 | 0.026 | -0.149 | 11222 |
| | tRNAs | 608 | 550 | 182 | 137 | 1,158 | 319 | 0.050 | -0.141 | 1477 |
| | rRNAs | 909 | 828 | 255 | 135 | 1,737 | 390 | 0.047 | -0.308 | 2127 |
| | A + T rich region | 770 | 735 | 123 | 63 | 1,505 | 186 | 0.023 | -0.323 | 1691 |
| *P. nitidula* | Whole genome | 7,181 | 6,698 | 1,993 | 1,480 | 13,879 | 3,473 | 0.035 | -0.148 | 17352 |
| | PCGs | 4,505 | 4,265 | 1,384 | 1,051 | 8,770 | 2,435 | 0.027 | -0.137 | 11205 |
| | tRNAs | 605 | 571 | 164 | 131 | 1,176 | 295 | 0.029 | -0.112 | 1471 |
| | rRNAs | 904 | 826 | 240 | 125 | 1,730 | 365 | 0.045 | -0.315 | 2095 |
| | A + T rich region | 1,061 | 969 | 195 | 168 | 2,030 | 363 | 0.045 | -0.074 | 2393 |
| *C. marinae* | Whole genome | 6,768 | 6,232 | 2,107 | 1,445 | 13,000 | 3,552 | 0.041 | -0.186 | 16552 |
| | PCGs | 5,015 | 4,674 | 1,697 | 1,211 | 9,689 | 2,908 | 0.035 | -0.167 | 12597 |
| | tRNAs | 589 | 565 | 169 | 144 | 1,154 | 313 | 0.021 | -0.080 | 1467 |
| | rRNAs | 921 | 797 | 255 | 129 | 1,718 | 384 | 0.072 | -0.328 | 2102 |
| | A + T rich region | 681 | 623 | 111 | 71 | 1,304 | 182 | 0.044 | -0.220 | 1486 |

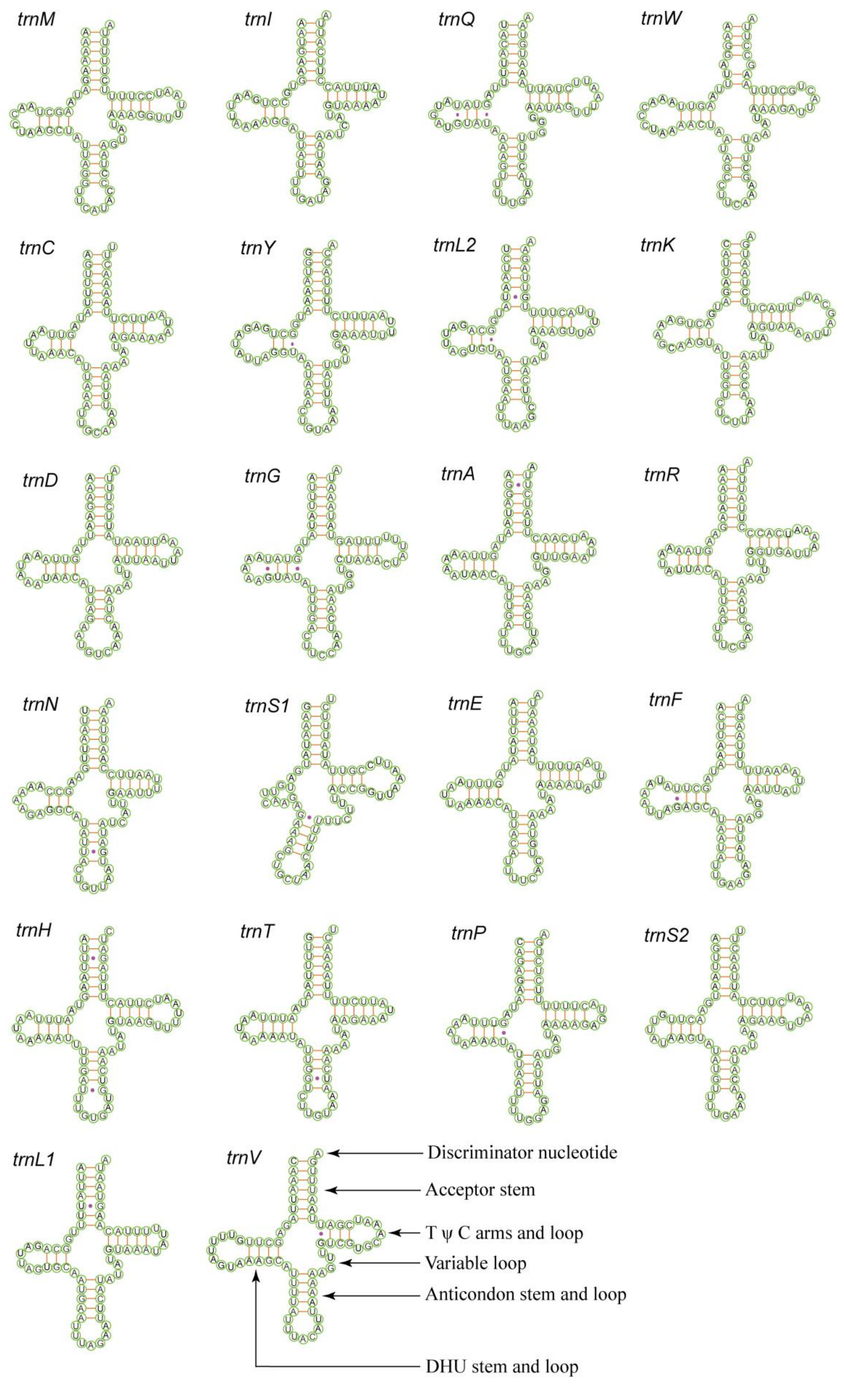


**Figure S1**: Secondary structure of tRNAs identified in the *Poecilus fortipes*

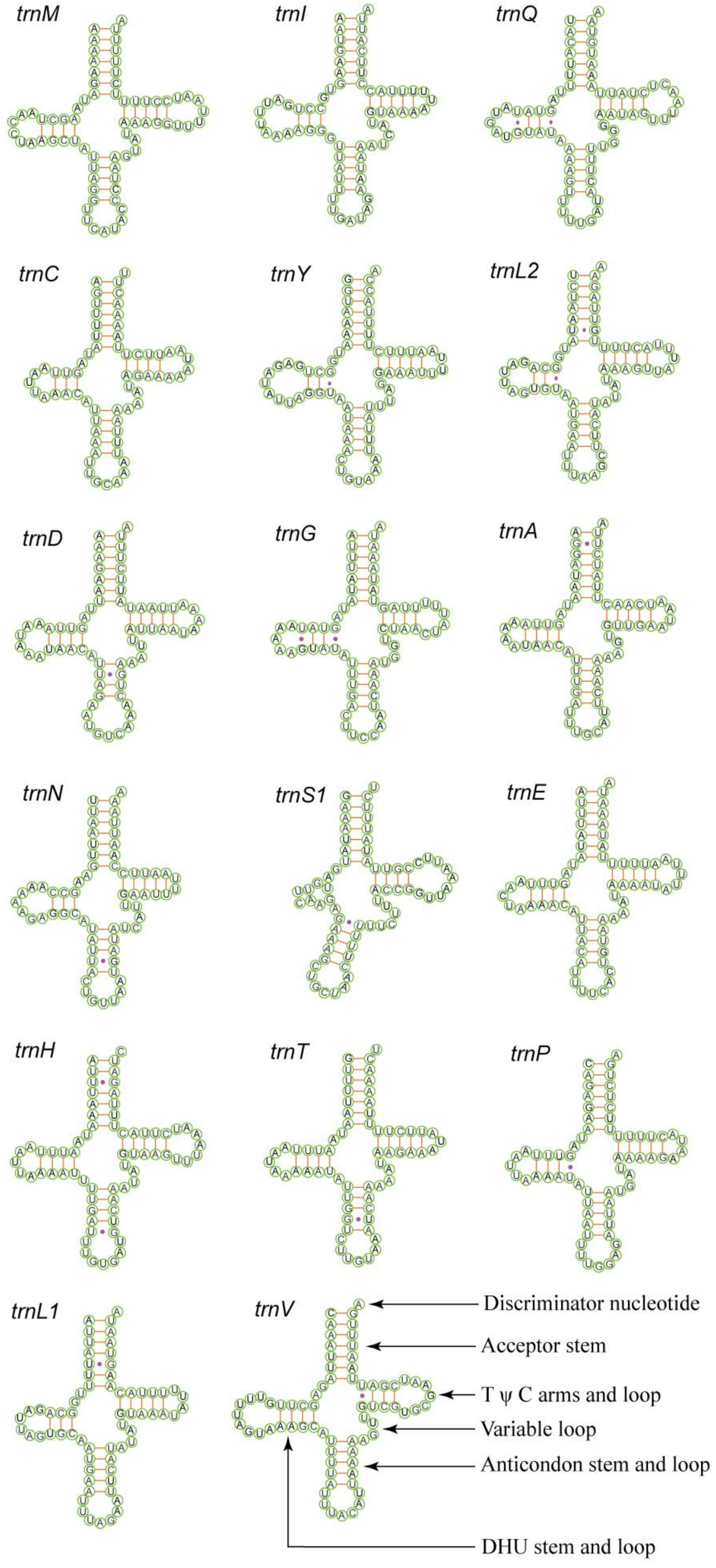


**Figure S2**: Secondary structure of tRNAs identified in the *Poecilus gebleri*

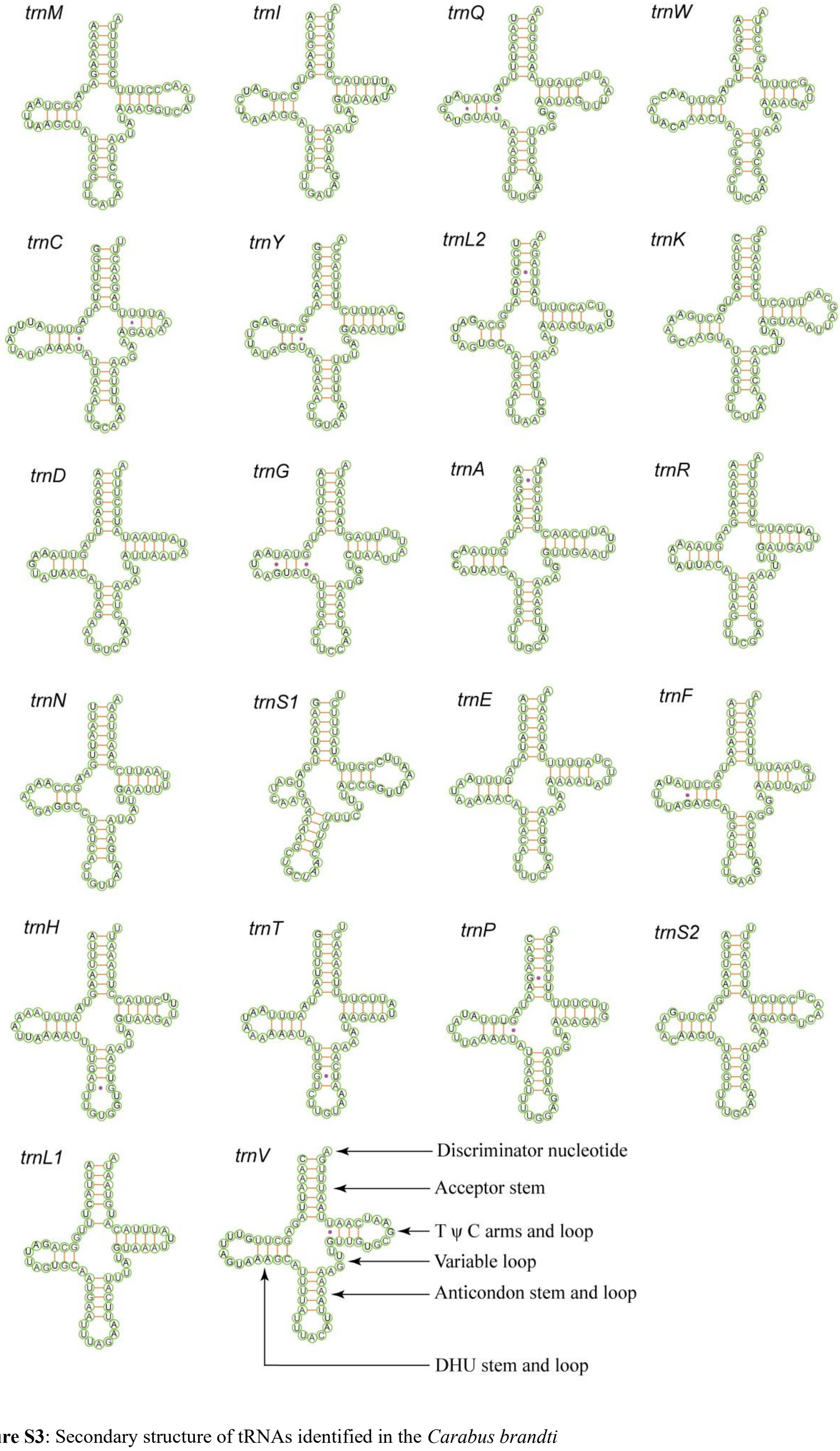


**Figure S3**: Secondary structure of tRNAs identified in the *Carabus brandti*

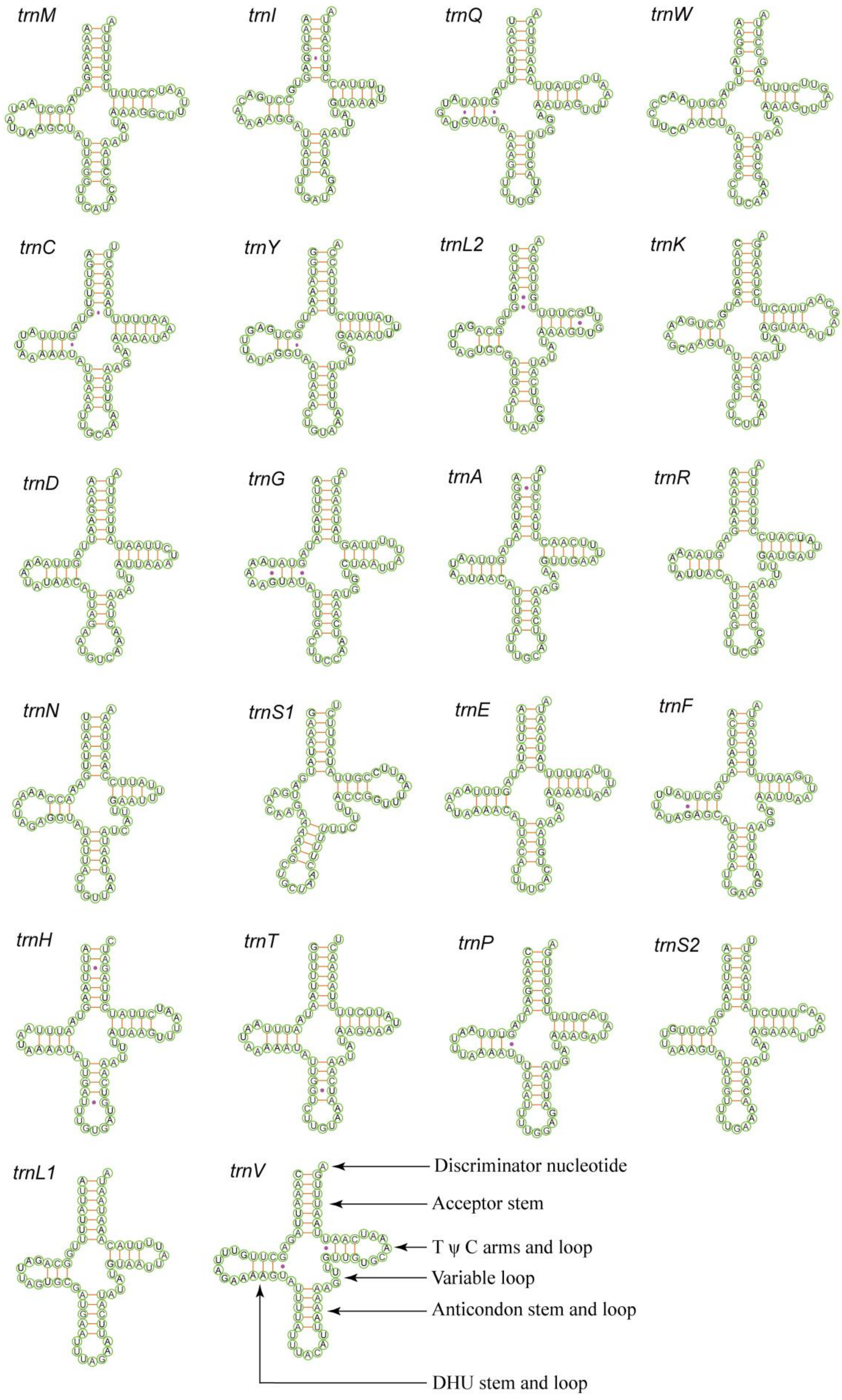


**Figure S4**: Secondary structure of tRNAs identified in the *Pristosia nitidula*

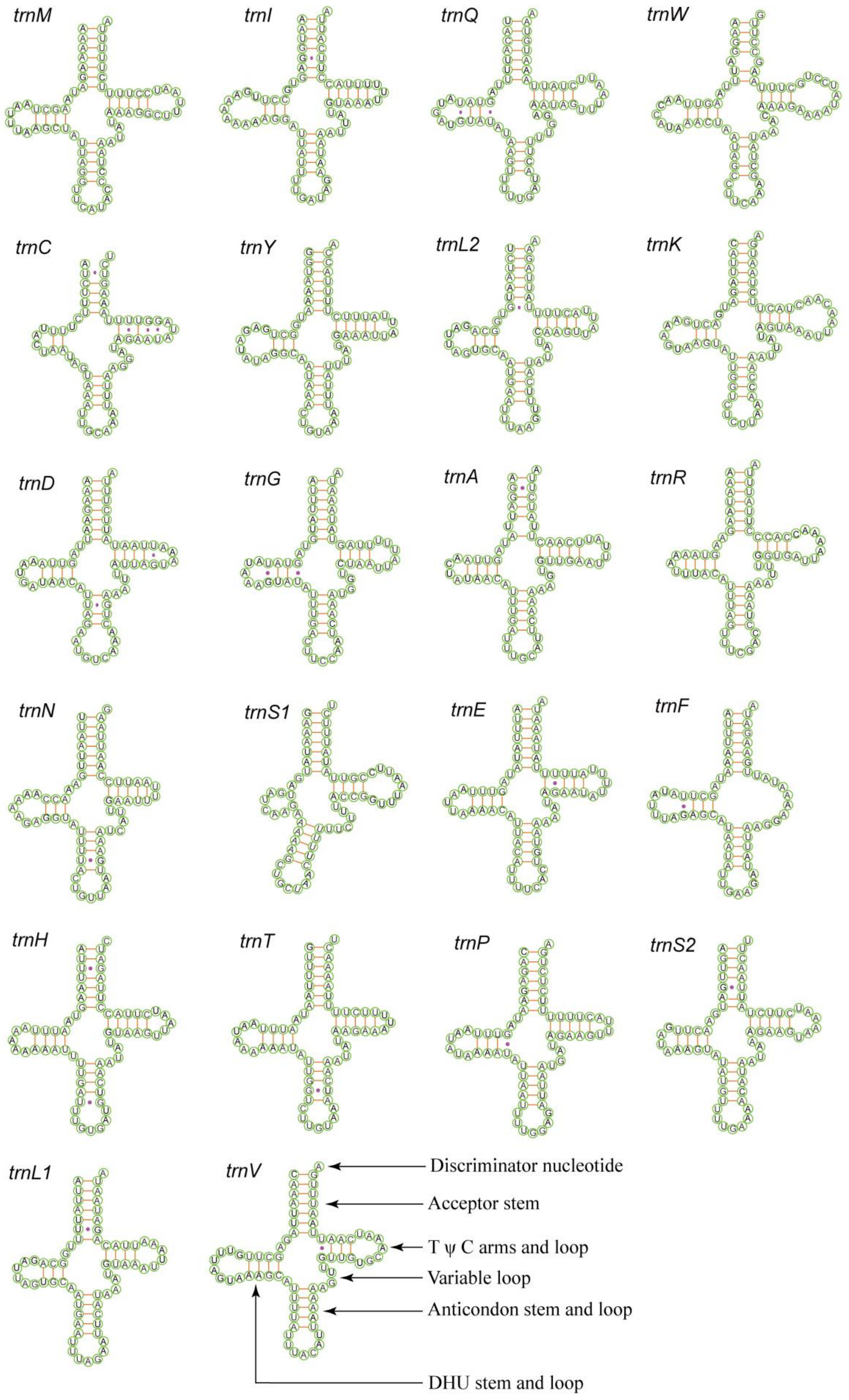


**Figure S5**: Secondary structure of tRNAs identified in the *Cephalosdrophus marinae*

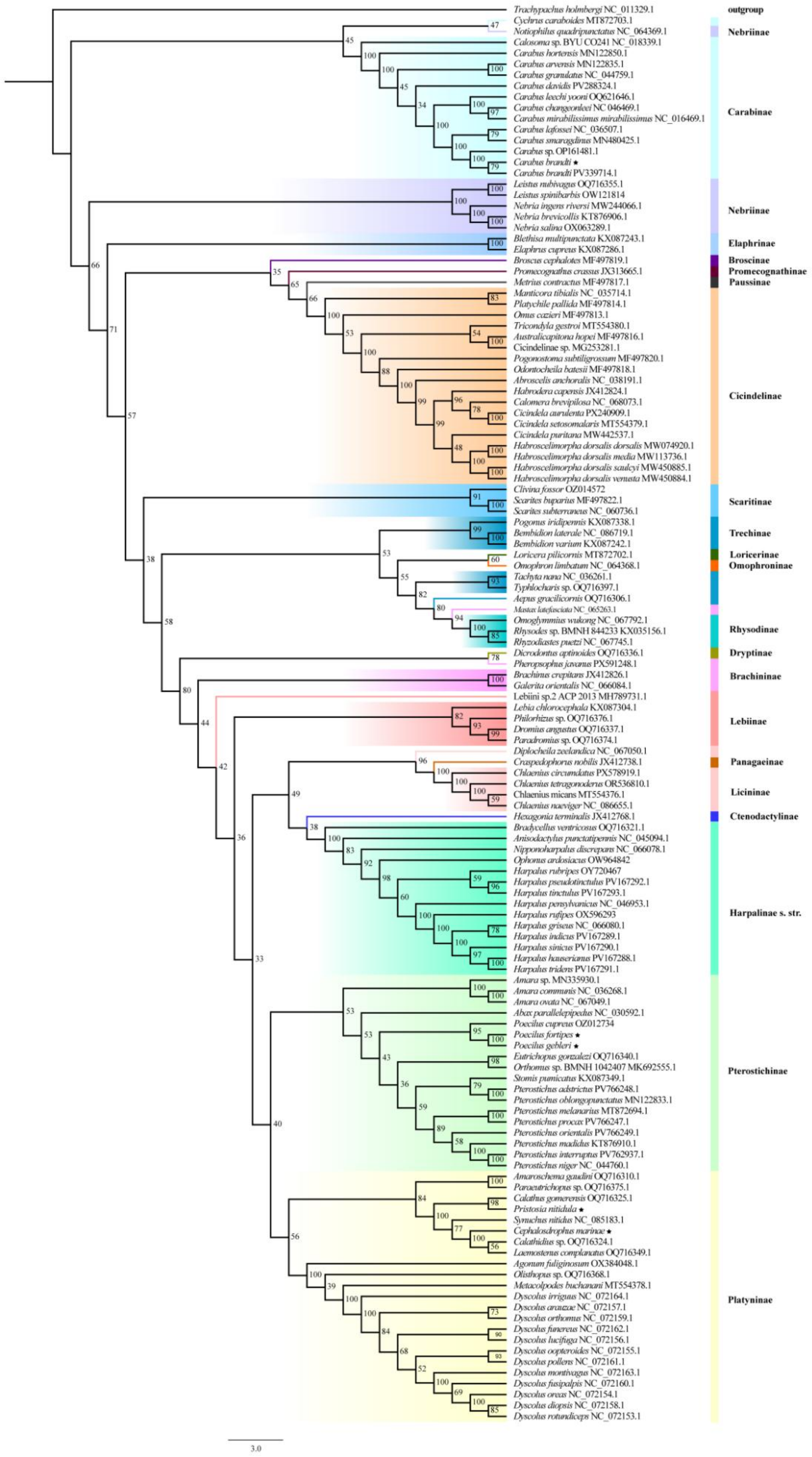


**Figure S6**: The maximum likelihood tree based on PCGsAA dataset. The numbers near the nodes are bootstrap support values (BS).

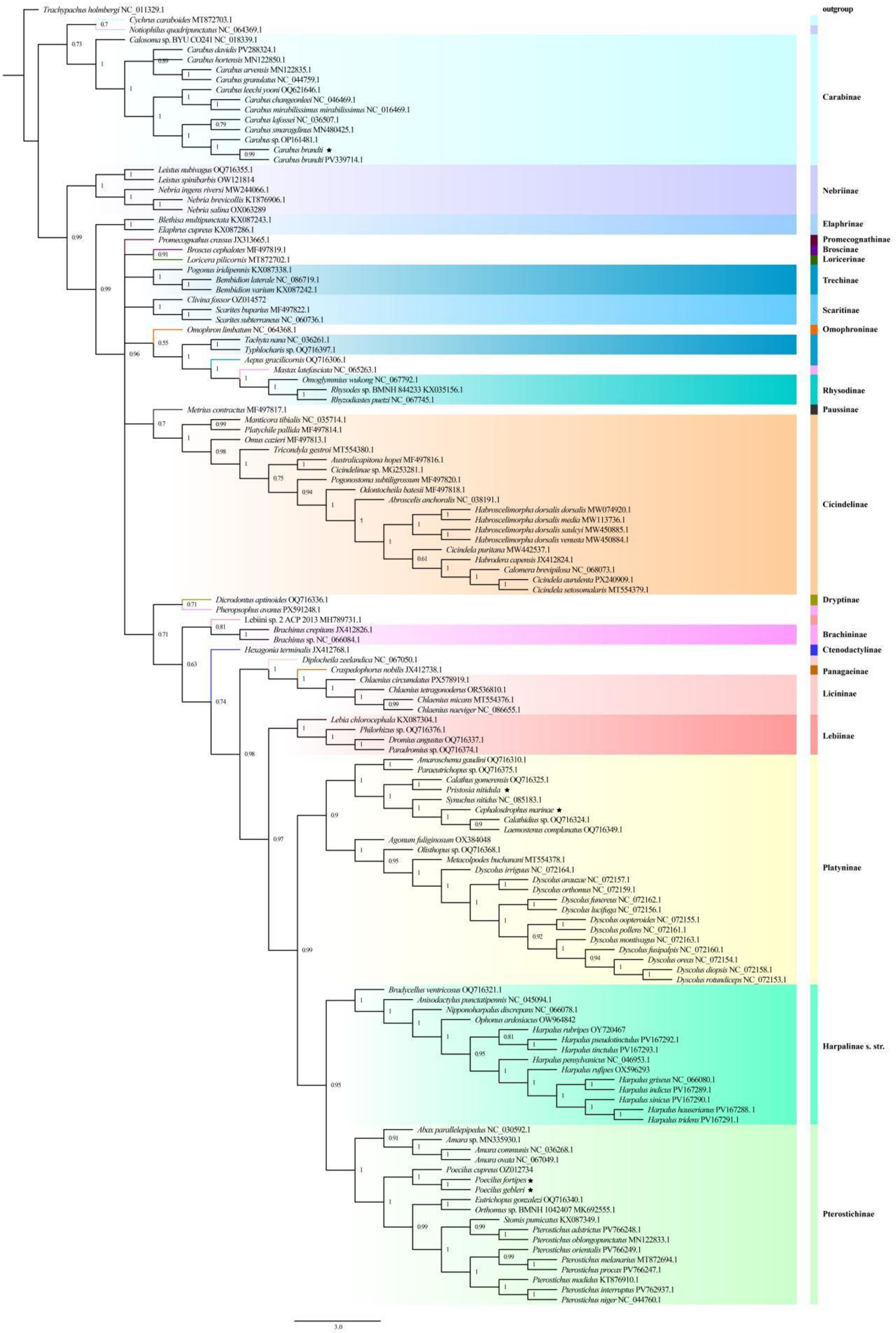


**Figure S7**: The Bayesian inference tree based on PCGsAA dataset. The numbers near the nodes are Bayesian posterior probabilities (PPs).

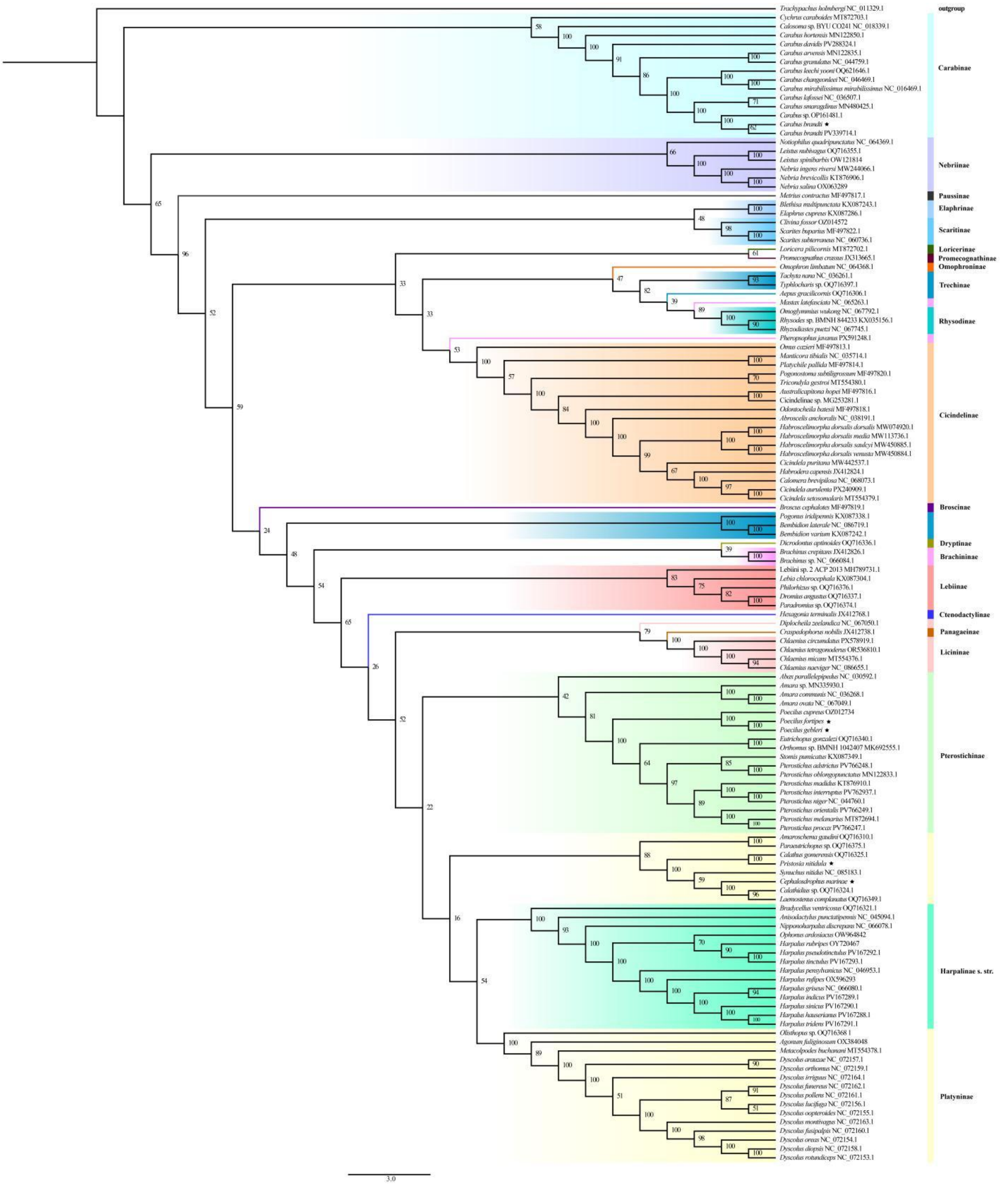


**Figure S8:** The maximum likelihood tree based on PCGsNT dataset. The numbers near the nodes are bootstrap support values (BS).

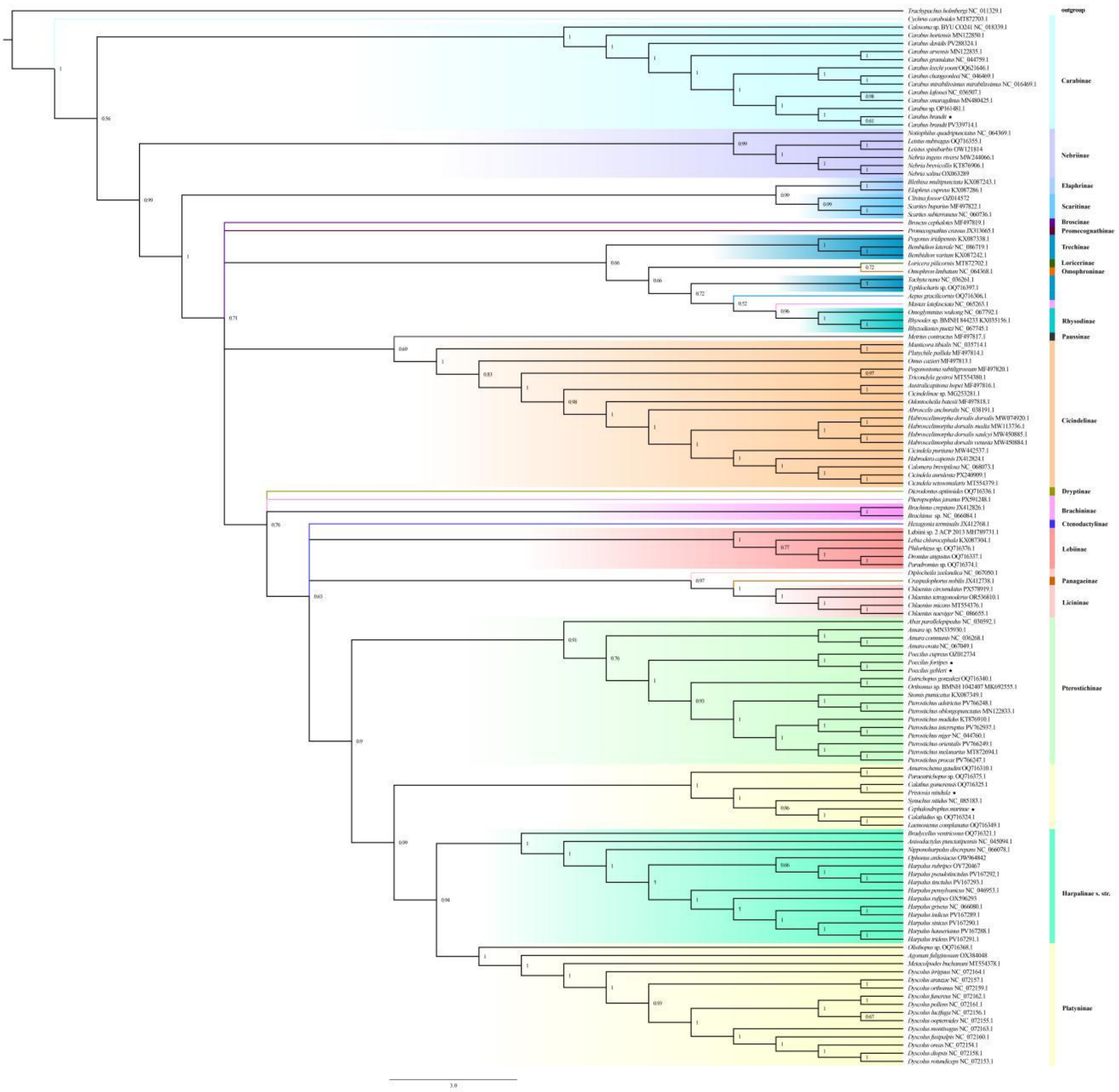


**Figure S9:** The Bayesian inference tree based on PCGsNT dataset. The numbers near the nodes are Bayesian posterior probabilities (PPs).

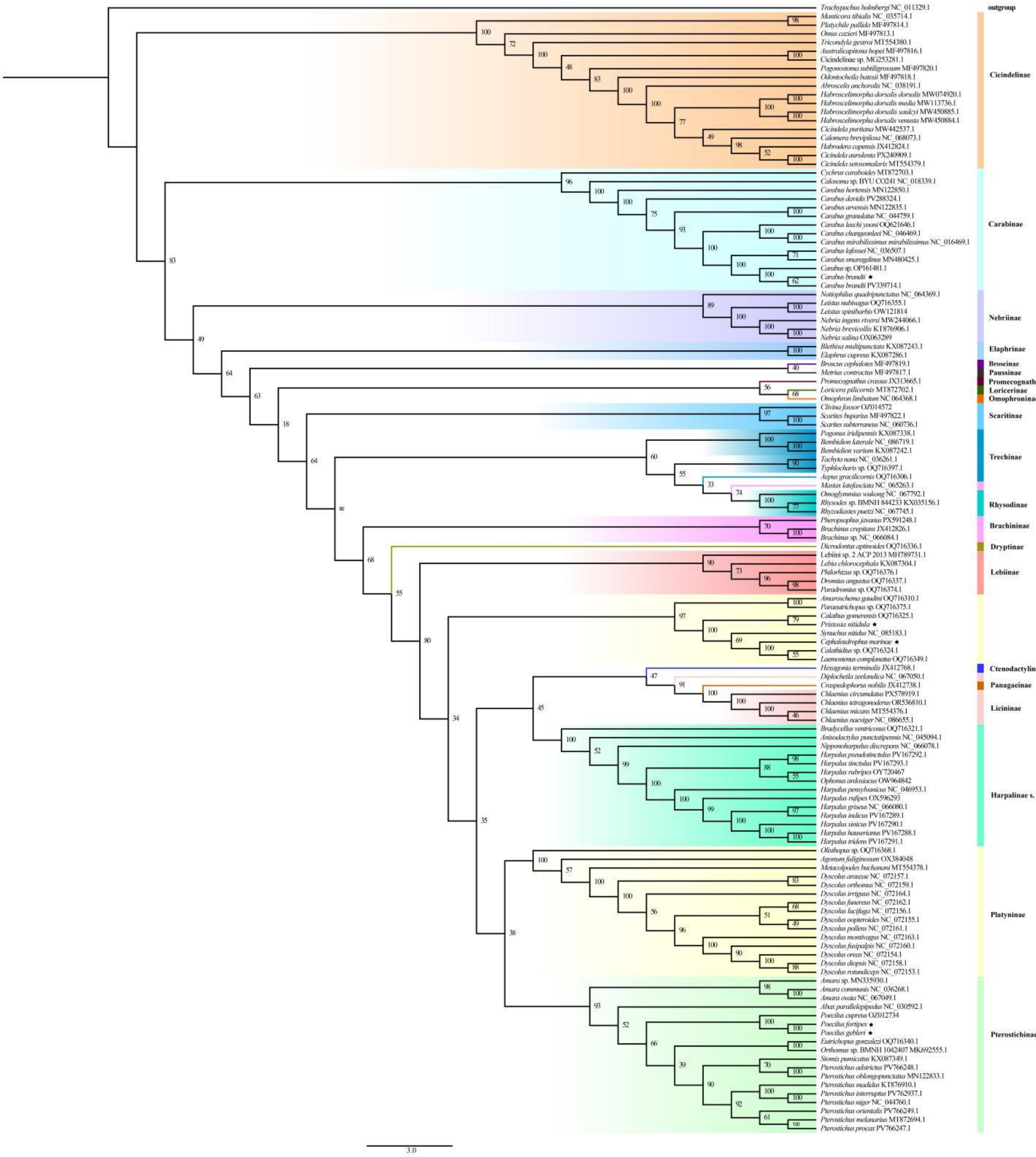


**Figure S10:** The maximum likelihood tree based on PCGs12R dataset. The numbers near the nodes are bootstrap support values (BS).

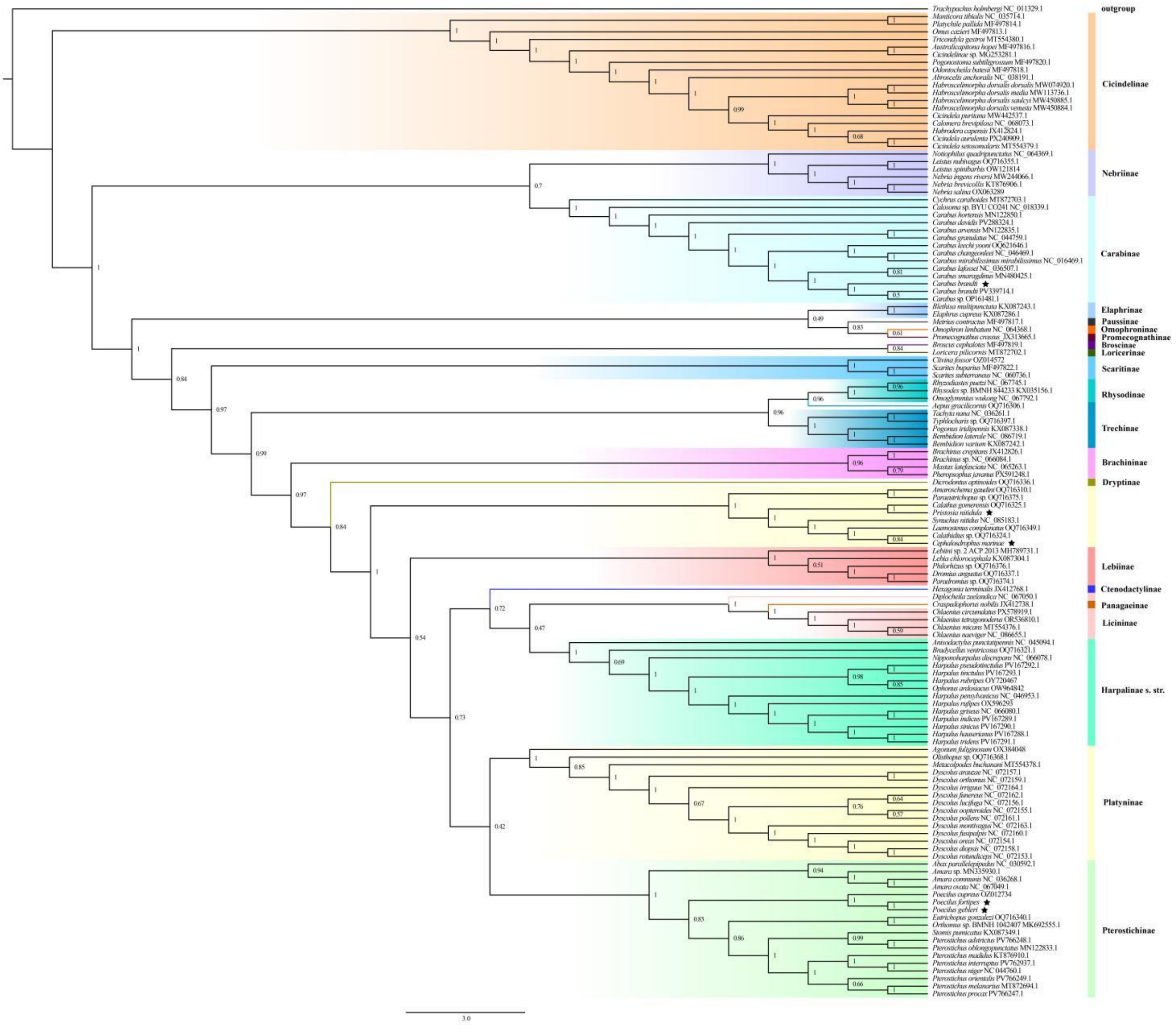

**Figure S11**: The Bayesian inference tree based on PCGs12R dataset. The numbers near the nodes are Bayesian posterior probabilities (PPs).

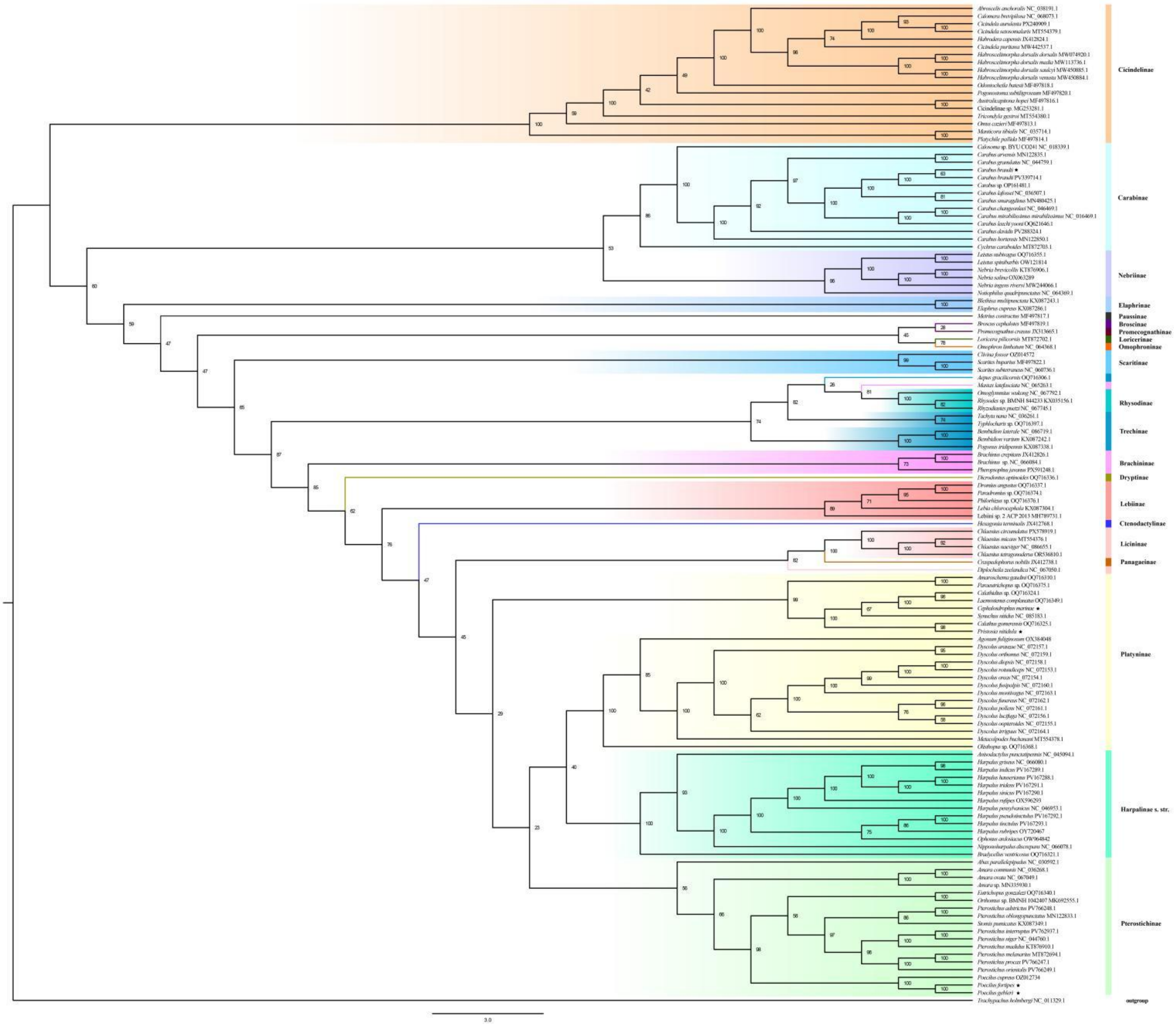


**Figure S12**: The maximum likelihood tree based on PCGs123R dataset. The numbers near the nodes are bootstrap support values (BS).
